# LncRNA HOXA11-AS promotes glioma malignant phenotypes and reduces its sensitivity to ROS via Tpl2-MEK1/2-ERK1/2 pathway

**DOI:** 10.1038/s41419-022-05393-5

**Published:** 2022-11-09

**Authors:** Cheng Wei, Xiaoyang Zhang, Dazhao Peng, Xu Zhang, Haizhen Guo, Yalin Lu, Lin Luo, Bo Wang, Zesheng Li, Yingjie He, Xuezhi Du, Shu Zhang, Hao Liang, Shenghui Li, Sheng Wang, Lei Han, Jianning Zhang

**Affiliations:** 1 Tianjin Neurological Institute, Key Laboratory of Post-Neuroinjury Neuro-repair and Regeneration in Central Nervous System, Ministry of Education and Tianjin City, Tianjin Medical University General Hospital, Tianjin, 300052 China; 2 School of Life Sciences, Tianjin University and Tianjin Engineering Center of Micro-Nano Biomaterials and Detection-Treatment Technology, Tianjin, 300072 China; 3 Department of Neurosurgery, The First Affiliated Hospital of Zhengzhou University, Zhengzhou, Henan 480082 China; 4 Department of Hepatopancreatobiliary Surgery, The Second Hospital of Tianjin Medical University, Tianjin, 300211 China

**Keywords:** CNS cancer, Long non-coding RNAs, Prognostic markers

## Abstract

Our previous studies showed that dysregulation of the long noncoding RNA (lncRNA) HOXA11-AS plays an important role in the development of glioma. However, the molecular mechanism of HOXA11-AS in glioma remains largely unknown. In this study, we explore the molecular mechanisms underlying abnormal expression and biological function of HOXA11-AS for identifying novel therapeutic targets in glioma. The expression of HOXA11-AS, and the relationship between HOXA11-AS and the prognosis of glioma patients were analyzed using databases and glioma samples. Transcriptomics, proteomics, RIP, ChIRP, luciferase, and ChIP assays were used to explore its upstream and downstream targets in glioma. The role of HOXA11-AS in regulating the sensitivity of glioma cells to reactive oxygen species (ROS) was also investigated in vitro and in vivo. We found that HOXA11-AS was significantly upregulated in glioma, and was correlated with the poor prognosis of glioma patients. Ectopic expression of HOXA11-AS promoted the proliferation, migration, and invasion of glioma cells in vitro and in vivo. Mechanistically, HOXA11-AS acted as a molecular sponge for let-7b-5p in the cytoplasm, antagonizing its ability to repress the expression of CTHRC1, which activates the β-catenin/c-Myc pathway. In addition, c-Myc was involved in HOXA11-AS dysregulation via binding to its promoter region to form a self-activating loop. HOXA11-AS, functioned as a scaffold in the nucleus, also recruited transcription factor c-Jun to the Tpl2 promoter, which activates the Tpl2-MEK1/2-ERK1/2 pathway to promote ROS resistance in glioma. Importantly, HOXA11-AS knockdown could sensitize glioma cells to ROS. Above, oncogenic HOXA11-AS upregulates CTHRC1 expression as a ceRNA by adsorbing let-7b-5p, which activates c-Myc to regulate itself transcription. HOXA11-AS knockdown promotes ROS sensitivity in glioma cells by regulating the Tpl2-MEK1/2-ERK1/2 axis, demonstrating that HOXA11-AS may be translated to increase ROS sensitivity therapeutically.

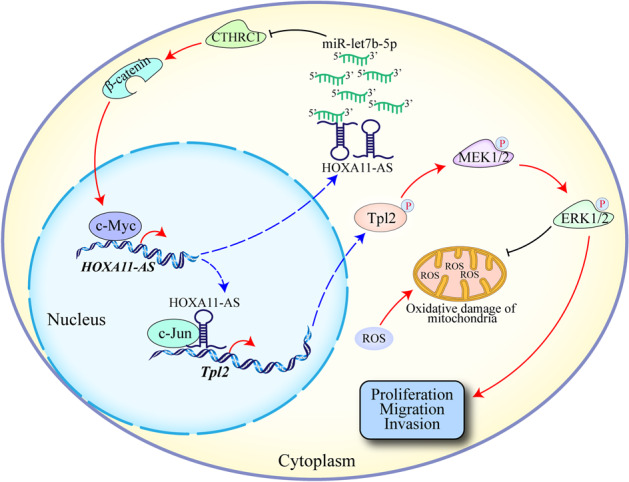

## Introduction

Malignant glioma is the most common primary intracranial tumor, the second leading cause of death among patients under the age of 34 years, and the third leading cause of death among patients aged 35–54 years [1]. The standard treatment is maximum safe surgical resection of the tumor, combined with postoperative chemotherapy and radiotherapy, but the therapeutic effect achieved is still unsatisfactory [2, 3]. The concept of reactive oxygen species (ROS) was first proposed in the 1950s and has gradually become a hot topic in cancer research [4]. Cancer cells have high ROS levels, and overexpress antioxidant enzymes to protect themselves from the consequent oxidative stress [5, 6]. Therefore, increasing the ROS levels in cancer cells, while inhibiting their antioxidant capacity to aggravate their oxidative stress, is a promising anticancer strategy [7–9].

Long noncoding RNAs (lncRNAs) are a class of noncoding RNAs more than 200 nucleotides (nt) in length [10]. Approximately 93% of the DNA in the human genome is transcribed into RNA, of which only 2% is protein-coding mRNA and the remaining 98% is noncoding RNA [11]. More than 28,000 lncRNAs have been identified in the human genome [12]. Recent studies have shown that lncRNAs are involved in epigenetic regulation, transcriptional regulation, post-transcriptional regulation, translation, post-translational modification, and chromosomal remodeling, by acting as guides, baits, scaffolds, and signal transducers [13, 14]. They are closely associated with the occurrence and progression of many major diseases, including cancer [15]. In addition, abnormal interaction between lncRNAs and signal transduction pathways might be the primary reason behind the resistance of glioma to ROS treatment.

In our previous studies, we noted a positive correlation between lncRNA HOXA11-AS (HOXA11 antisense RNA) expression, and the grade of glioma patients. HOXA11-AS could regulate glioma cell cycle progression, and maintain the tumor cell stemness [16]. There is accumulating evidence that HOXA11-AS can promote the proliferation, migration, and invasion of a variety of tumor cells through molecular scaffolds, molecular sponges, and other mechanisms [17, 18]. However, there are few studies on the molecular mechanism of HOXA11-AS in glioma.

In this research, we studied the expression of HOXA11-AS in glioma using online databases and glioma samples, and analyzed the relationship between HOXA11-AS expression and the prognosis of glioma patients. The biological functions of HOXA11-AS in glioma have been verified in vitro and in vivo. Through mRNA and small RNA sequencing, the ectopic expression mechanism of HOXA11-AS in glioma was explored. In addition, we determined that HOXA11-AS acts as a key regulator of glioma sensitivity to ROS through transcriptomics, proteomics, and ROS sensitivity assays. Finally, using ROS-producing nanoparticles (NPs), we demonstrated that HOXA11-AS could regulate the ROS sensitivity of glioma cells in vitro and in vivo. Our findings revealed the potential of HOXA11-AS as a therapeutic target to mediate ROS sensitivity in glioma.

## Material and methods

### Data collection and processing

The clinical data and RNA sequencing data about 33 tumors and corresponding normal tissues were downloaded from TCGA and GTEx databases via UCSC Xena (https://xenabrowser.net/datapages/). In the analysis of glioma alone, 255 cases of normal brain tissue from GTEx database and 529 cases of low-grade glioma (LGG, WHO II-III), and 173 cases of glioblastoma (GBM, WHO IV) from TCGA database were included. The online database, GEPIA2 (http://gepia2.cancer-pku.cn/#index), was used for prognosis analysis of patients with multiple tumors [19]. The HOXA11-AS mRNA expression profiles in four subtypes (Classical, Mesenchymal, Neural, and Proneural) and the related prognoses of the glioma patients were analyzed through four databases (CGGA, TCGA, REMBRANDT, and GSE16011). Based on median values of RNA expression levels, glioma patients were classified into high and low-expression groups, and survival curves and log-rank tests were used for validation. The subcellular localization of HOXA11-AS in different cell lines was predicted in the lncATLAS database (https://lncatlas.crg.eu/) [20].

The COX analysis of HOXA11-AS in glioma from TCGA database was analyzed on SangerBox portal (http://SangerBox.com/Tool). Analysis of HOXA11-AS related genes in CGGA database was performed using R package. The positively or negatively correlated genes with HOXA11-AS expression were screened in CGGA database. TCGA dataset was used to screen HOXA11-AS positively (Top 50) or negatively (Top50) correlated genes in LinkedOmics (http://www.linkedomics.org/login.php). SangerBox portal was used to analyze and map the results of GO and KEGG analyses. Transcription factors that could bind to the Tpl2 promoter region were predicted via the PROMO database. The c-Jun binding peaks of Tpl2 promoter region and c-Myc binding peaks of HOXA11-AS promoter region were predicted via UCSC portal. The binding of c-Jun to the Tpl2 promoter region was predicted via Tartaglialab website (http://www.tartaglialab.com/).

### Tumor specimens and cell culture

All of the primary human glioma specimens were obtained from patients who underwent surgery at the First Affiliated Hospital of Zhengzhou University, and each sample contained 80% tumor cells, as confirmed by microscopic examination. The tissue samples were graded by the neuropathologist in accordance with the World Health Organization (WHO) criteria and stored in liquid nitrogen. The glioma specimens included grade II (17 samples), grade III (8 samples), and grade IV (16 samples). This study was approved by the institutional review boards of the hospitals, and written informed consent was obtained from all patients. The histology and clinical data of the glioma samples are available in Supplementary Table 1.

The human astrocytes, U87, LN229, U251, TG905, and LN308 cell lines, which were all cultured in the Tianjin Neurological Institute of Tianjin Medical University General Hospital, and mycoplasma detection and STR cell identification were carried out. The human astrocytes were grown in AM medium (Astrocyte Medium, Sciencell, USA). The U87, LN229, U251, and LN308 were grown in Dulbecco’s modified Eagle’s medium (DMEM) supplemented with 10% fetal bovine serum (FBS, BI serum, Israel). The TJ905 cell line were cultured with DMEM/F12 medium (Gibco, USA). Two patient-derived primary GBM cells (primary GBM cell 1 and primary GBM cell 2) were obtained from glioma tissues surgically removed from WHO grade IV glioma patients in Tianjin Medical University General Hospital, which were approved by the ethics committee, and informed consent was signed. Cells were grown in serum-free medium (DMEM-F12/Neurobasal [1:1 mix] with 1% B27 and 2 mM L-glutamine and supplemented with 20 ng/mL each of eEGF and FGF2). All cells were grown at 37 °C in a humidified atmosphere (95% humidity) with 5% CO_2_.

### Lentivirus infection and plasmids transfection

The lentivirus carrying the HOXA11-AS cDNA sequence (Lv-HOXA11-AS) were used to upregulate HOXA11-AS expression in U87 (MOI = 5) and LN229 (MOI = 10) cell lines, and control lentivirus (Lv-NC) was used to construct control cell lines. In addition, the lentivirus carrying the HOXA11-AS shRNA sequence (Lv-shHOXA11-AS) were used to knockdown HOXA11-AS expression in U87 (MOI = 5) and LN229 (MOI = 10) cell lines, and control lentivirus (Lv-NC) was used to construct control cell lines. Both Lv-NC, Lv-HOXA11-AS, and Lv-shHOXA11-AS were purchased from Genechem Co., Ltd. (Shanghai, China).

The pGL3-Tpl2 plasmid was purchased from Hanbio Biotechnology Co., Ltd. (Tianjin, China). The pGL3-Tpl2 plasmid was used to upregulate Tpl2 expression. The pcDNA3.1-HOXA11-AS plasmid was purchased from Genewiz (Beijing, China) and used to upregulate HOXA11-AS expression. The c-Jun plasmids (pcDNA3.1-Flag-Jun-full length (FL), pcDNA3.1-Flag-Jun-TAD, pcDNA3.1-Flag-Jun-241-254, and pcDNA3.1-Flag-Jun-DBD) were purchased from Genewiz (Beijing, China) (The schematic diagram of c-Jun plasmids was shown in Fig. 5D). The plasmids were transfected with Lipofectamine 3000 (L3000015, Thermo Fisher Scientific, USA) according to the manufacturer’s instructions. The above plasmids were confirmed by agarose gel electrophoresis and DNA sequence analysis. The overexpression efficiency was detected by qPCR and western blotting as described below.

### SiRNA and miRNA mimics/inhibitors transfection

The siRNAs, let-7b-5p mimics, and inhibitors used in this study were all purchased from GenePharma Co., Ltd. (Shanghai, China). SiRNAs and let-7b-5p mimics/inhibitors were transfected into cell lines using Lipofectamine RNAiMax (13778150, Thermo Fisher Scientific, USA) according to the manufacturer’s instructions. The siRNAs and let-7b-5p mimics/inhibitors sequences were shown in Supplementary Tables 2 and 3. The knockdown or overexpression efficiency was detected by qPCR and western blotting as described below.

### RNA isolation, reverse transcription (RT), and quantitative real-time PCR

Total RNA was extracted using TRIzol reagent (Takara, Japan). The mRNA and lncRNA (3 µg) were reverse transcribed for the synthesis of cDNA using a GoScript Reverse Transcription system (A5001, Promega, USA). The miRNA RT assay was conducted using the Hairpin-it microRNA and U6 snRNA Normalization RT-PCR Quantitation Kit (GenePharma, China). The expression status of mRNA and lncRNA were measured on an ABI QuantStudio 3 using GoTaq® qPCR Master Mix (A6001, Promega, USA), and the expression of GAPDH was used as an internal control. In addition, the expression status of miRNA was measured on an ABI QuantStudio 3 using Hairpin-it microRNA and U6 snRNA Normalization RT-PCR Quantitation Kit, and the expression of U6 was used as an internal control. For mRNA and lncRNA, the PCR system was 20 μL, and the following procedures were performed: 40 cycles were performed at 95 °C for 3 min, 95 °C for 15 s, and 60 °C for 1 min. The dissolution curve program is: 95 °C 15 s, 60 °C 1 min, 95 °C 1 s. For miRNA, the following procedures were performed: 40 cycles were performed at 95 °C for 3 min, 95 °C for 12 s, and 62 °C for 40 s. The relative expression level of the target gene was calculated by 2^−△△CT^. The primer sequences showed in Supplementary Table 4.

### CCK-8 and EdU assay

The cells to be detected were seeded into 96-well plates in advance, generally ranging from 2 × 10^3^ to 1 × 10^4^ cells. The test was started 24 h later and lasted for 5 consecutive days. The medium in the well was absorbed and discarded, and CCK-8 reagent (APExBIO, USA) was mixed with serum-free medium in a 5 ml tube in advance and added into 96-well plates with 100 μL per well (CCK8 reagent: Serum-free medium = 10 μL: 90 μL per well). After incubation at 37 °C for 1 h, OD value was detected on spectrophotometric measurements at 450 nm. EdU assay Kit (Cell-Light EDU Apollo488 In Vitro Kit) was purchased from Ribobio Co., Ltd. (Guangzhou, China). The experiment was carried out in strict accordance with the kit instructions.

### Wound healing and Transwell experiments

For wound healing experiment: according to the density of 2 × 10^5^ cells per well, U87 and LN229 cells were inoculated in the 6-well plate. After 24 h, siRNA transfection was carried out. After 24 h, 200 μL pipetting head was used to scratch the cells in the plate, and the serum-free medium was changed. Images at 0 h, 12 h, and 24 h were collected under an inverted microscope (IX81, Olympus Company, Japan) and the cell migration rate was calculated and analyzed using ImageJ software. Each assay was replicated three times.

For Transwell experiment: the upper chamber of Transwell was coated with a layer of Matrigel matrix glue (matrix glue: serum-free medium = 1:4 v/v ratio) (Corning, USA). After solidification for 40 min at 37 °C in cell culture incubator, the upper chamber was placed in a 24-well plate, and the cells were resuspended and counted using serum-free medium. 5 × 10^4^ cells were seeded into the upper chamber with 200 μL serum-free medium, and the lower chamber was added with 500 μL of full medium containing 10% fetal bovine serum. After 24 h of culture, 4% paraformaldehyde was used to fixate cells for 10 min, 1% crystal violet was used to staining cells for 10 s, the remaining cells in the upper chamber were slightly wiped off, and images were collected under a positive microscope (BX53, Olympus Company, Japan) and the number of gelled cells was counted. Each assay was replicated three times.

### Immunofluorescence assay

Cell slides were made in advance, and 2 × 10^4^ to 5 × 10^4^ cells were planted on each 24-well cell slide. After 24 h, the medium was discarded, and the cells were fixed with 4% formaldehyde for 15 min. PBS was used to wash cells for three times, 3 min each time. 0.5% Triton X-100 was used to incubate for 20 min at room temperature, and the cells was washed with PBS for 3 times, 3 min each time. PBS was absorbed and dried with absorbent paper. Then, a sufficient amount of diluted β-catenin (CST, 8480, 1:100) or Alexa FluorTM 594 phalloidin (A12381, Thermo Fisher Scientific, USA) was added to each well. The solution was placed into a cartridge and incubated overnight at 4 °C. The cells were washed with PBST for 3 times, 3 min each. The diluted fluorescent secondary antibody was added and incubated for 1 h in 37 °C without light. The cells were washed with PBST for 3 times, 3 min each time. Each hole was dyed with 100 μL DAPI working solution for 10 min, and cleaned with PBST for 4 times, 5 min each time. The cover glass was dried with absorbent paper and laced upside down on the slide with anti-fluorescence attenuation sealing agent (Sigma, USA), then the fluorescence image was collected.

### Isolation of nuclear and cytoplasmic RNA

After the cells were prepared, the medium was absorbed and discarded, washed twice with PBS, and the cells were scraped off with 400 μL precooled PBS. Then, transferred into 1.5 ml tube, centrifuged at 10,000 rpm at 4 °C for 10 s, and the supernatant was discarded. Resuspend the cells with 400 μL PBS containing RNA enzyme inhibitor (N2615, Promega, USA) (RNA enzyme inhibitors: PBS = 1:20 v/v ratio) and 0.1% NP-40 (85124, Thermo Fisher Scientific, USA) (NP-40: PBS = 1:1000 v/v ratio), put it in ice for 5 min, then vortexed for 5 s. After centrifugation at 10,000 rpm at 4 °C for 20 s, the supernatant was extracted from cytoplasm and transferred into a new tube. At this time, the supernatant was cytoplasm extract, and the precipitate was nucleus. The nuclei were resuspended by 200 μL PBS containing 0.5% NP-40 and RNA enzyme inhibitor (RNA enzyme inhibitors: PBS = 1:20 v/v ratio), and were blown for 10 times, placed on ice for 10 min, and centrifuged for 20 s at 13,000 rpm at 4 °C. The supernatant was the nuclear extract, and nuclear extract and cytoplasmic extract were used for subsequent RT-qPCR experiments according to the above steps.

### RNA fluorescence in situ hybridization (RNA-FISH)

GFP-labeled HOXA11-AS probes were obtained from GenePharma Co., Ltd. (Shanghai, China). Hybridizations were carried out using FISH Kit (GenePharma) according to the manufacturer’s instructions. 4% paraformaldehyde was used to fix the glioma cells followed by the treatment of 0.5% Triton. Then, cells were cultured with specific probe overnight. All fluorescence images were captured using IX81 fluorescence inverted microscope (Olympus, Japan). The sequence for HOXA11-AS probe is: 5′-AATGCGAGACTCCAGGAGAATGCGGATCAGTGACAAACCGGAG GAGGGAGTTTCTCCAGAGGCTGTGGAAAGAAGCGTA-3′.

### Molecular docking, RNA immunoprecipitation (RIP), and Chromatin isolation by RNA purification (ChIRP)

The 3D structure of Ago2 was from https://www.rcsb.org/. The HOXA11-AS model was generated from the MC-Fold/MC-Sym program and analyzed for energy optimization using TINKER. Subsequently, we executed molecular docking between Ago2 and HOXA11-AS employing the SYBYL-X 2.0 program.

The Magna RIP™ RNA-Binding Protein Immunoprecipitation Kit (17-700, Merck Millipore, USA) was used according to the manufacturer’s instructions. In c-Jun or Ago2 RIP experiments, 4 × 10^7^ U87 cells were harvested and lysed in RIP lysis buffer. Each lysate was further divided into three groups for anti-c-Jun (anti-Ago2 or anti-Flag), anti-IgG (negative control), and Input (positive control). Either c-Jun, Ago2, Flag, or IgG antibody was added to each sample to enrich RNA binding protein (RBP). Subsequently, the RBP of interest, together with the bound RNA, were collected using dynabeads. After washing off unbound material, the RBP was digested by Proteinase K, and the RNA bound to immunoprecipitated RBP was purified and reverse-transcribed into cDNA. Then, qPCR assay was performed to measure the % Input of HOXA11-AS in each group. The primer sequences and antibody information used for RIP analysis were provided in Supplementary Tables 4 and 5.

For ChIRP assay, ChIRP Kit (BersinBio, China) was used to verify the binding of HOXA11-AS to let-7b-5p and all the experimental steps were carried out in accordance with the kit instructions. A 3′ end Biotin modified-DNA probe targeting HOXA11-AS was synthesized and provided by BersinBio. 1.2 × 10^8^ glioma cells were cross-linked with 1% formaldehyde and sonicated for the hybridization reaction. After the chromatin was sheared into 100–500 bp fragments, the cell lysates were incubated with the biotinylated DNA probe solution for 3 h at 37 °C. The binding complex was covered with streptavidin-conjugated magnet beads. RNA was finally eluted and purified from the magnet beads for RT-qPCR analysis. The sequences of the probes are available in Supplementary Table 6.

### Luciferase reporter assay

The sequences of HOXA11-AS cDNA targeted by let-7b-5p were inserted into the upstream of the firefly luciferase in the pSICHECK2 vector to obtain pSICHECK2-HOXA11-AS reporter plasmid (Fig. 2H) and CTHRC1 3′-UTR were inserted into the upstream of the firefly luciferase in the pSICHECK2 vector to obtain pSICHECK2-CTHRC1 reporter plasmid (Hanbio, China) (Fig. 3B). The Tpl2 luciferase reporter plasmid (pTpl2-luc) was purchased from Hanbio Biotechnology Co., Ltd. (Tianjin, China) in order to detect the transcriptional activity of Tpl2. The c-Myc luciferase plasmid (pMyc-TA-luc) were purchased from Beyotime Biotechnology (Shanghai, China) to detect the transcriptional activity of c-Myc. The TOP/FOP luciferase reporter plasmids were obtained from Merck Millipore (USA) to detect the transcriptional activity of β-catenin. The promoter sequence of Tpl2, including A (−500/0), B (−1000/0), C (−1500/0), and D (-2000/0), were PCR-amplified from the genomic DNA of U87 cells, which were then inserted into the HindIII-NheI sites upstream of the firefly luciferase in the pGL3-Basic vector (Genewiz, China) (Fig. 5F). The luciferase plasmids were transfected with Lipofectamine 3000 and luciferase activity was measured with a Bright-Glo™ Luciferase Assay System (E2620, Promega, USA) according to the instructions. The luciferase expression was detected with a microplate reader (Synergy2, BioTek, USA).

### Western blot

The cells were lysed by RIPA lysis buffer (Solarbio, China) containing a protease and phosphatase inhibitor cocktail (Sigma) and the protein concentration was detected with a BCA kit (Solarbio, China) according to the manufacturer’s instructions. Proteins were separated by 10% SDS-PAGE gels and transferred with 0.22 μm PVDF membranes (Millipore, USA). The membranes were blocked and incubated with specific antibodies overnight at 4 °C. The membranes were then incubated with the corresponding HRP-conjugated secondary antibody. The protein bands were visualized and detected by the enhanced chemiluminescence system (Bio-Rad, Hercules, EDA USA). The relevant antibodies used are shown in Supplementary Table 5.

### Chromatin immunoprecipitation (ChIP) assay

U87 and LN229 cells were cultured to 90% confluence. Cells were harvested for chromatin immunoprecipitation (ChIP) by ChiP kit (56383, CST, USA), according to manufacturer’s protocols. First, solubilized chromatin was prepared from a total of 2 × 10^7^ cells. The chromatin solution was diluted 10‐fold with ChIP dilution buffer and precleared with protein A beads and preimmune serum. The precleared chromatin solution was divided and utilized in immunoprecipitation assays with anti‐c-Jun, anti-c-Myc, or anti‐IgG antibody. Following, the antibody‐protein‐DNA complex was eluted from the beads. After cross‐linking, protein and RNA were removed and the purified DNA was subjected to PCR with primers specific for the Tpl2 promoter region containing the c-Jun binding sites or HOXA11-AS promoter region containing the c-Myc binding sites. The primer sequences and antibody information were shown in Supplementary Tables 5 and 7.

### Transcriptomics and proteomics analysis

U87 cells infected with Lv-NC or Lv-HOXA11-AS were used for transcriptomics and proteomics. Based on the TMT labeling-based proteomics, we comparatively quantified the host proteome of Lv-NC and Lv-HOXA11-AS cells. Each group contained 3 duplicate samples. The transcriptomic analysis (including mRNA and small RNA sequencing) in this study was conducted at Gene Denovo Biotechnology Co., Ltd. (Guangzhou, China), and the proteomic analysis was conducted at Zhongke New Life Biotechnology Co., Ltd (Shanghai, China). The criteria for determining differential genes in transcriptomics were |fold change|(FC) > 1.5, *P* < 0.05. The proteomic criteria were |fold change|>1.2, *P* < 0.05 (Fig. 4A).

### Determination of mRNA half-life

To assess the half-life of Tpl2 mRNA, actinomycin-D (Act D, Sigma, A4262) was used to block mRNA synthesis. The Lv-HOXA11-AS were infected into U87 and LN229 cells. After 24 h, ActD was added to the culture medium, followed by incubation for 0 h, 2 h, 4 h, 6 h, 8 h, and 12 h. Total RNA was collected at different time points and Tpl2 mRNA stability in the ActD treatment group was analyzed by qPCR.

### Reactive oxygen species (ROS) assay

U87 and LN229 cells were transfected with either si-HOXA11-AS or si-NC. After 48 h, cells were collected and cell lysates were prepared. Cellular ROS level were measured by fluorescence plate reader using Reactive Oxygen Species Assay Kit (Solarbio, China) according to the manufacturer’s instructions.

For oxidative stress treatment in vitro assay, H_2_O_2_ was purchased from Sigma (29.4 μmol/μL, USA). Firstly, 10,000 cells were planted in 96-well plates per well in order to detect the IC50 (half maximal inhibitory concentration) of H_2_O_2_ in U87 and LN229 cell lines. The absorbance (OD) of each well was measured by CCK-8 at 8 h after dosing and the survival rate and IC50 of different concentration groups was calculated. The pH-sensitive nanoparticles (NPs) and pyropheophorbide-a (PPa) labeled NPs were prepared according our previous reports [21, 22]. In brief, methyl linoleate hydroperoxide (MLH) (40 μL, 100 mM in N,N-Dimethylformamide) was mixed with a Tetrahydrofuran (THF) solution of poly (ethylene glycol)-block-poly (diisopropylaminoethyl methacrylate) (PEG-PDPA) (3 mL, 8 mg). The MLH and PEG-PDPA were prepared by ourselves [21]. After dropwise addition of 8 mL of pure water, the solution containing nanomedicine was evaporated to remove organic solvent. To prepare PPa-labeled NPs, PEG-PDPA (4 mg) and PEG-PDPA-PPa (4 mg) were added during preparation. In addition, dynamic light scattering (DLS) experiment were performed with a Zetasizer Nano instrument (Malvern Instruments Ltd., United Kingdom) equipped with a 10-mW helium-neon laser (λ = 632.8 nm) and thermoelectric temperature controller to detect the particle size of NPs. The IC50 of NPs was measured in U87, LN229, and primary GBM cells at 24 h, 48 h, and 72 h after the addition of NPs and calculated at each time point using spectrophotometer.

### Subcutaneous xenograft model and intracranial tumor model construction

All animal procedures were conducted in accordance with protocols approved by the Tianjin Medical University Animal Care and Use Committee and followed guidelines for animal welfare. Four-week-old BALB/c female nude mice were purchased from Beijing HFK Bioscience Co., LTD. For subcutaneous xenograft models, 5 × 10^5^ cells were suspended in 50 µL of PBS and implanted into the flanks of nude mice. For siRNA-mediated knockdown of HOXA11-AS in vivo, when the tumor volume reached 100 mm^3^, the animals were randomized into three groups with 7 mice in each group: si-NC group, si-HOXA11-AS#1 group and si-HOXA11-AS#2 group. For siRNA-mediated knockdown of Tpl2 in vivo, when the tumor volume reached 100 mm^3^, the animals were randomized into two groups with 10 mice in each group: si-NC and si-Tpl2 groups. After that, tumor volumes were measured every 2 days and si-HOXA11-AS or si-Tpl2 were injected into the knockdown group at a dose of 10 μL of siRNA versus 10 μL of Lipofectamine ^TM^ 3000 per nude mouse. After 17 or 21 days, the nude mice were sacrificed, subcutaneous tumors were removed, images were collected. The tumor volume was calculated with the formula Volume = (length × width^2^)/2.

For biodistribution assay, U87 cells were infected with lentiviruses of GFP-luciferase (GenePharma, China) to establish a stable GFP-luciferase (GFP-luc) overexpression GBM cell model. 5 × 10^5^ U87-GFP-luc cells were injected into the intracranial striatum of nude mice with a stereotactic instrument and a microinfusion pump (Stoelting Co., USA). And orthotopic glioma-bearing mice implanted with U87-GFP-luc cells were randomly divided into two groups (4 mice per group) and received one-time i.v. injection of Free-PPa or PPa-NPs (all at a dose equivalent of 4.0 mg/kg Free-PPa or PPa-NPs per mouse). The fluorescence intensity of the Free-PPa and PPa-NPs groups were monitored by the assistance of fluorescence imaging on hours 4, 12, 24, 48, and 72 via the IVIS imaging system (perkinelmer, USA) after injection of Free-PPa or PPa-NPs. After 72 h, the major organs were removed for fluorescence imaging again, and then the brain tissue was fixed, dehydrated, and sectioned into 10-μm slices for IF.

For in vivo therapeutic model, shRNA lentiviruses (or scrambled lentivirus, GenePharma, China) were used to construct U87 cell lines with HOXA11-AS knockdown stably. 5 × 10^5^ control or HOXA11-AS knockdown U87 cells were injected into the intracranial striatum of nude mice with a stereotactic instrument and a microinfusion pump (Stoelting Co., USA). The animals were divided into 4 groups (control, NPs, Lv-shHOXA11-AS, Lv-shHOXA11-AS + NPs), with 10 mice in each group. Starting on day 14 after tumor cell implantation, NPs (13.33 μmol/kg, iv) was given every other day for 4 times. Other animals in control and Lv-shHOXA11-AS groups were treated with an equal volume of PBS alone. In order to obtain tumor growth status in live animals of different treatment groups by bioluminescent imaging, the mice were anesthetized and injected intraperitoneally with D-luciferin (150 mg/kg, beetle luciferin, potassium salt, E1605, Promega) 10 min prior to imaging with the IVIS imaging system (PerkinElmer, USA) for 10–120 s. Eight weeks post implantation, the surviving nude mice in each group were sacrificed. Survival analysis was determined using Kaplan–Meier survival curve. The major organs were collected and sectioned into 10-μm slices for hematoxylin and eosin (H&E) and immunohistochemistry (IHC) staining.

### Hematoxylin and eosin (HE)staining, immunohistochemistry (IHC) staining and Immunofluorescence (IF) assay

In order to conduct histological analysis, tumor tissues were fixed in 10% neutral buffered formalin for HE staining and IHC analysis. For HE staining, the HE staining kit (Solarbio, China) was used according to the manufacturer’s instructions. Pictures were taken using an Olympus upright BX53 microscope. For IHC analysis, 8 µm slides were dewaxed in xylene and then rehydrated through graded alcohols to distilled water (dH_2_O). Antigen retrieval was performed using sodium citrate (pH=6) buffer at 97 °C for 20 min. After washing in PBS, slides were blocked with 5% blocking serum for 30 min at room temperature. Next, the slides were incubated at 4 °C overnight with primary antibodies against β-catenin, Tpl2, p-MEK, and p-ERK before being incubated with a biotin-labeled secondary antibody (1:100 dilution) for 1 h at 37 °C, followed by incubation with diaminobenzidine (DAB). The slides were then counterstained with hematoxylin and mounted. Pictures were taken using an Olympus upright BX53 microscope. For IF assay, brain tissues were fixed in 4% paraformaldehyde and then dehydrated in 15% and 30% sucrose before optimal cutting temperature (OCT) compound embedding and cutting into 10 µm slides. After washing in PBS, slides were blocked with 3% blocking serum (contain 0.3% Triton X-) for 60 min at room temperature. The slides were incubated at 4 °C overnight with primary antibodies against NeuN. Then, the diluted fluorescent secondary antibody was added and incubated for 1 h in room temperature without light. Each slide was dyed with 100 μL DAPI working solution for 10 min, and cleaned with PBST for 3 times, 10 min each time. The cover glass was dried with absorbent paper and laced upside down on the slide with anti-fluorescence attenuation sealing agent (Sigma, USA), then the fluorescence image was collected by an Olympus FV1200 laser scanning confocal microscope. Antibodies information are shown in Supplementary Table 5.

### Statistical analysis

The image processing software used in this study was Photoshop CS6, and the image production and statistical calculation software was GraphPad Prism 8. In this study, unpaired *t*-test was used for the difference comparison between the two groups involved, one-way ANOVA was used for the comparison between the multiple groups, and two-way ANOVA was used for the comparison of OD values between the multiple groups of CCK-8. The log-rank test was used for survival analysis. *P* < 0.05 was considered statistically significant.

## Results

### The clinical and genetic features of HOXA11-AS in glioma

**Fig. 1:**
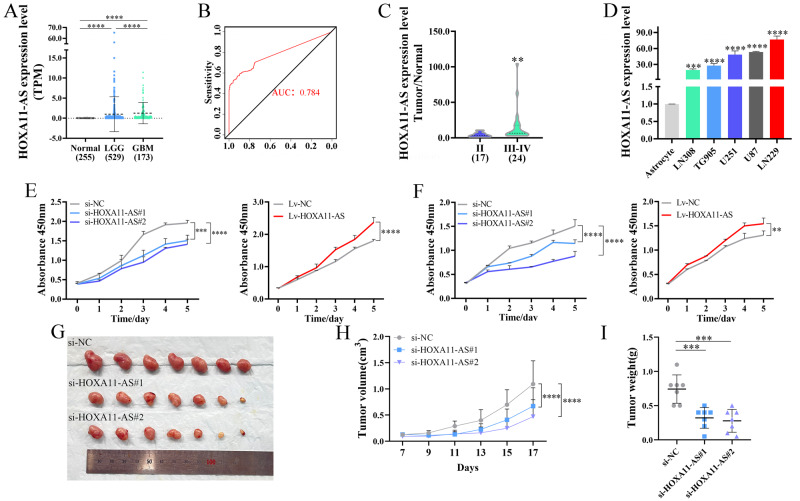
Clinical features and biological functions of HOXA11-AS in glioma. **A** The expression levels of HOXA11-AS in normal brain tissue, LGG (WHO II-III), and GBM in TCGA and GTEx databases. **B** ROC curve shows the difference of HOXA11-AS expression in normal brain tissue and glioma from TCGA and GTEx databases (AUC = 0.784). **C** The expression levels of HOXA11-AS in 41 glioma samples, including WHO II (*n* = 17) and WHO III-IV (*n* = 24). **D** The expression levels of HOXA11-AS in astrocyte, LN308, TG905, U251, U87, and LN229 cells were verified by RT-qPCR. The effect of HOXA11-AS overexpression or knockdown on proliferation by CCK8 experiment in U87 (**E**) and LN229 cells (**F**). Xenograft mouse models were generated by subcutaneous injection of U87 cells into nude mice. Seven days after implanted, mice were treated with si-NC (*n* = 7), si-HOXA11-AS#1 (*n* = 7), and si-HOXA11-AS#2 (*n* = 7). Representative image of dissected tumors (**G**), tumor growth curve (**H**) and tumor weight (**I**), in si-NC, si-HOXA11-AS#1 and si-HOXA11-AS#2 treated xenograft models with U87 cells. **P < 0.01, ***P < 0.001, ****P < 0.0001.

Our previous studies showed a positive correlation between lncRNA HOXA11-AS (HOXA11 antisense RNA) expression and the grade of glioma patients. Moreover, HOXA11-AS could regulate glioma cell cycle progression, and maintain the tumor cell stemness [16]. Then, we analyzed the expression and prognosis of HOXA11-AS in glioma patients. Analysis of the TCGA and GTEx databases revealed that the expression levels of HOXA11-AS in glioblastoma (GBM) were higher than those in low-grade glioma (LGG, WHO II-III) and normal tissues (Fig. 1A). The receiver operating characteristic (ROC) curve also indicated that the expression of HOXA11-AS differed between normal brain tissue and glioma (Fig. 1B). To validate these findings, we performed real-time quantitative PCR (RT-qPCR) analysis of RNA expression in 41 glioma samples and found much higher levels of HOXA11-AS in WHO III-IV grade than that in WHO II grade (Fig. 1C). The subtype analyses were applied in CGGA, TCGA, REMBRANDT, and GSE16011 databases, and results showed that HOXA11-AS expression was closely associated with subtypes of glioma patients in CGGA, which may be related to the race (Fig. S1A–D). The analyses of CGGA, TCGA, REMBRANDT, and GSE16011 databases also revealed that the expression level of HOXA11-AS was closely related to the prognosis of glioma (Fig. S1E–H). Cox regression analysis in TCGA database showed that HOXA11-AS was an independent prognostic factor for glioma. (Fig. S1I–J).

Furthermore, we further analyzed HOXA11-AS expression levels and prognosis in multiple tumors. The TCGA and GTEx databases showed elevated levels of HOXA11-AS in 16 tumor types, compared to their corresponding normal tissues (Fig. S2A). Prognostic analysis using the GEPIA database also indicated that high HOXA11-AS levels in a variety of tumors, including adrenocortical cancer (ACC), kidney papillary cell carcinoma (KIRP), LGG, and pancreatic cancer (PAAD), were associated with poor overall survival (OS) and recurrence-free survival (RFS) (Fig. S2B–C). These findings suggest that HOXA11-AS is an oncogenic lncRNA associated with poor prognosis in multiple tumors, including glioma. Therefore, we focused on HOXA11-AS and investigated its biological functions and molecular mechanisms in glioma.

### The biological functions of HOXA11-AS

The expression of HOXA11-AS was examined in a panel of glioma cell lines via RT-qPCR, and the U87 and LN229 GBM cell lines were selected for subsequent experiments because of their elevated levels of HOXA11-AS (Fig. 1D). We knocked down and overexpressed HOXA11-AS in these two cell lines using siRNA and lentivirus of HOXA11-AS, respectively (Fig. S3A–B). CCK-8 and EdU assays showed that HOXA11-AS knockdown significantly inhibited cell proliferation, whereas its ectopic expression promoted cell growth (Figs. 1E–F and S4A–D). In addition, overexpression of HOXA11-AS promoted cell migration (Fig. S4E–F) and invasion (Fig. S4G–H) whereas its depletion reduced cell migration (Fig. S5A–B) and invasion (Fig. S5C–D). HOXA11-AS also enhanced the formation of filopodia in glioma cells (Fig. S5E–F).

Next, we investigated the effects of HOXA11-AS knockdown on glioma growth in vivo. Xenograft mouse models were generated by the subcutaneous injection of U87 cells into nude mice. Seven days after implantation, the mice were treated with scramble siRNA or si-HOXA11-AS (Two siRNAs), respectively. The experiment was terminated after 17 days of treatment. The mice in si-HOXA11-AS#1 or si-HOXA11-AS#2 treatment group showed much lower tumor growth rate, smaller tumor size, and lighter tumor weight, as compared to those of the mice in the si-NC group (Fig. 1G–I). Collectively, these results indicate that elevated HOXA11-AS levels promote glioma cell proliferation, migration, invasion, and tumor growth.

### HOXA11-AS is a competitive endogenous RNA (ceRNA) of let-7b-5p

**Fig. 2:**
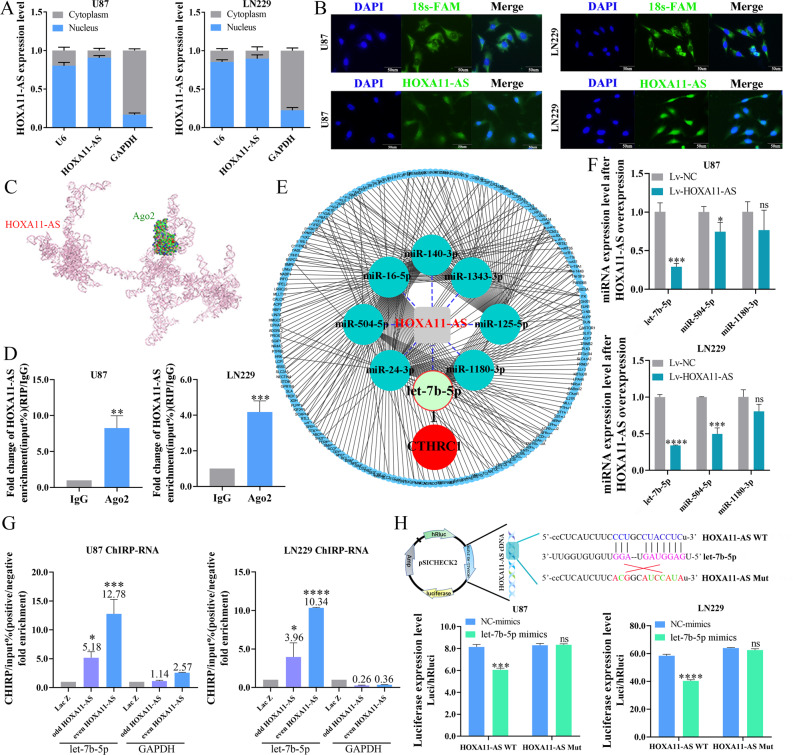
Exploration about the ceRNA mechanism of HOXA11-AS in cytoplasm. The subcellular localization of HOXA11-AS was verified by RT-qPCR (**A**) and FISH (**B**) experiments in U87 and LN229 cell lines. **C** The binding mode of HOXA11-AS to Ago2 protein was predicted via molecular docking analysis. **D** The binding of HOXA11-AS to Ago2 protein was verified by RIP experiment. **E** The ceRNA network diagram was constructed by mRNA and small RNA sequencing after HOXA11-AS overexpression in U87 cell line. **F** The expression of let-7b-5p, miR-504-5p, and miR-1180-3p in U87 and LN229 cells infected with Lv-HOXA11-AS were quantified by RT-qPCR. **G** The combination of HOXA11-AS and let-7b-5p was verified by ChIRP assay. **H** Luciferase reporter assay in U87 and LN229 cells co-transfected with let-7b-5p mimics, pSICHECK2-wild type HOXA11-AS (WT) or pSICHECK2-mutant type HOXA11-AS (Mut) plasmids. *P < 0.05, **P < 0.01, ***P < 0.001, ****P < 0.0001.

To define the molecular mechanisms of HOXA11-AS in glioma, we initially examined the cellular localization of HOXA11-AS in glioma. Analysis of the LncATLAS database indicated that HOXA11-AS was primarily localized in the nucleus (Fig. S6A), and RT-qPCR and FISH experiments on U87 and U251 cells revealed that HOXA11-AS was partially localized in the cytoplasm, but primarily localized in the nucleus (Fig. 2A–B), implying that it functions in both the cytoplasm and the nucleus. Previous studies have shown that many of the cytoplasmic lncRNAs act as molecular sponges (ceRNAs). Therefore, we next investigated whether cytoplasmic HOXA11-AS functions as a ceRNA in glioma.

Molecular docking analysis predicted that HOXA11-AS binds to the Ago2 protein (Fig. 2C), which acts as the catalytic engine of the RNA-induced silencing complex (RISC) and plays a significant role in miRNA guided post-transcriptional gene silencing. RNA immunoprecipitation (RIP) analysis further revealed that HOXA11-AS forms a complex with Ago2 (Fig. 2D). Next, we performed mRNA and small RNA sequencing in U87 cells infected with Lv-HOXA11-AS or Lv-NC and found significant differential expression of 615 mRNAs (404 upregulated and 211 downregulated mRNAs in Lv-HOXA11-AS-infected cells) and 83 miRNAs (14 upregulated and 69 downregulated miRNAs in Lv-HOXA11-AS-infected cells), between Lv-HOXA11-AS- and Lv-NC-infected U87 cells (Fig. S6B–D). Then, using association analysis, we constructed a ceRNA network of HOXA11-AS in glioma to identify its target miRNAs, which included let-7b-5p, miR-504-5p, miR-1180-3p, miR-16-5p, miR-24-3p, miR-125b-5p, miR-140-3p, and miR-1343-3p (Fig. 2E). The CGGA database search showed reduced expression of let-7b-5p, miR-504-5p and miR-1180-3p in HGG compared with LGG, with poor overall survival (Fig. S6E–K). Since HOXA11-AS levels were elevated in HGG, we next investigated whether HOXA11-AS is the ceRNA of let-7b-5p, miR-504-5p, and miR-1180-3p. RT-qPCR showed that let-7b-5p expression was mostly decreased in response to the ectopic expression of HOXA11-AS (Fig. 2F). The ChIRP assay revealed that let-7b-5p binds to HOXA11-AS (Figs. 2G and S7A–B). HOXA11-AS was co-localized with let-7b-5p in U87 and LN229 cells (Fig. S7C). Furthermore, dual-luciferase assay using U87 and LN229 cells indicated that HOXA11-AS is a direct target of let-7b-5p (Fig. 2H). These results indicate that HOXA11-AS serves as a ceRNA for let-7b-5p in glioma.

### CTHRC1 is a direct target of HOXA11-AS/let-7b-5p

**Fig. 3:**
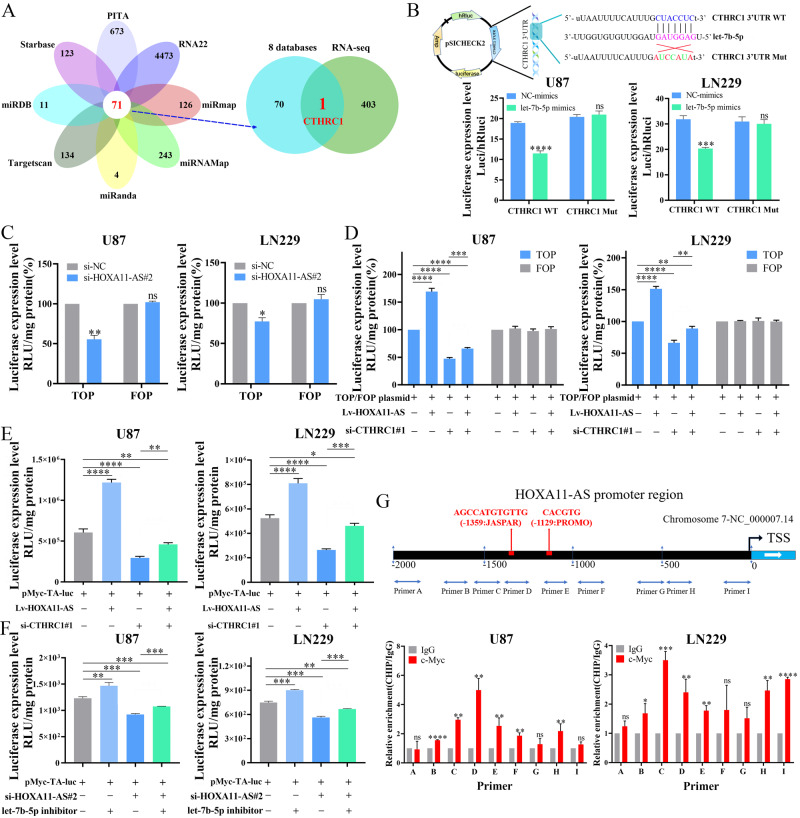
HOXA11-AS can mediate its own transcription via regulating let-7b-5p/CTHRC1/β-catenin/c-Myc axis. **A** Venn diagram showing the predicted target genes of let-7b-5p. **B** U87 and LN229 cells were co-transfected with reporter vectors containing the wild-type CTHRC1 3′UTR (CTHRC1 WT) or CTHRC1 3′UTR with a mutated let-7b-5p binding site (CTHRC1 Mut) and either let-7b-5p mimics or scramble oligonucleotide (NC-mimics). **C** The effect of HOXA11-AS knockdown on β-catenin transcriptional activity was detected by TOP/FOP assay. **D** HOXA11-AS regulated the transcriptional activity of β-catenin depending on CTHRC1 via TOP/FOP assay. **E** HOXA11-AS regulating the transcriptional activity of c-Myc depending on CTHRC1 was demonstrated via luciferase assay. **F** HOXA11-AS regulating the transcriptional activity of c-Myc depending on let-7b-5p was verified via luciferase assay. **G** The binding sites of c-Myc bound to the HOXA11-AS promoter region were verified via ChIP assay. *P < 0.05, **P < 0.01, ***P < 0.001, ****P < 0.0001.

As the HOXA11-AS/let-7b-5p axis was active in glioma, we next explored its downstream targets. Analyses of eight databases (PITA, RNA22, miRmap, miRNAMap, miRanda, TargetScan, miRDB, and Starbase) revealed 71 mRNAs as possible downstream targets of let-7b-5p (Fig. 3A, left). In comparison with our mRNA sequence results, which showed 404 upregulated mRNAs in Lv-HOXA11-AS-infected U87 cells, we found that only CTHRC1, a critical gene promoting tumor progression and metastasis including glioma, overlapped with the let-7b-5p target set (Fig. 3A, right). Thus, we examined whether CTHRC1 was the downstream target of HOXA11-AS/let-7b-5p.

Our previous studies showed that CTHRC1 was overexpressed in various cancer types and functions as an important oncogene that may promote tumorigenesis and development through different mechanisms including glioma [23]. Analysis of CTHRC1 expression in 41 glioma samples confirmed these results (Fig. S8A). RT-qPCR revealed that CTHRC1 expression dramatically decreased after transfection of let-7b-5p mimics into U87 and LN229 cells (Fig. S8B–D). To further demonstrate the direct regulation of CTHRC1 by let-7b-5p, we constructed luciferase reporter plasmid system with the targeting sequences of wild-type and mutated CTHRC1-3′UTRs (Fig. 3B, top), and transfected them into U87 and LN229 cells. Luciferase activities of wild-type, but not mutant CTHRC1 luciferase plasmid, were significantly suppressed by let-7b-5p mimics (Fig. 3B, bottom).

Since HOXA11-AS acts as a ceRNA for let-7b-5p, we observed a significant increase in CTHRC1 mRNA levels following the ectopic expression of HOXA11-AS (Fig. S9A). RT-qPCR analysis of 41 glioma samples revealed a positive correlation between HOXA11-AS expression and CTHRC1 expression (Fig. S9B). In addition, we demonstrated that the overexpression of HOXA11-AS reversed the inhibitory effect of let-7b-5p mimics on CTHRC1 expression (Fig. S9C–D). These results indicated that CTHRC1 is a direct target of HOXA11-AS/let-7b-5p.

### HOXA11-AS regulates the β-catenin/c-Myc pathway through let-7b-5p/CTHRC1 to form a unique self-activation loop

CTHRC1 activates β-catenin pathway by inducing its nuclear localization [24, 25]. As HOXA11-AS increases CTHRC1 expression by serving as a ceRNA for let-7b-5p, we next investigated whether HOXA11-AS regulates the β-catenin pathway. Cell fractionation and immunofluorescence staining showed that ectopic expression of HOXA11-AS promoted the translocation of β-catenin from the cytoplasm into the nucleus (Fig. S9E–F). As β-catenin is a co-activator of TCF/LEF transcription factors that initiate the transcription of various genes, including c-Myc, cyclin D1, and c-Jun, we further determined whether CTHRC1 regulates c-Myc transcription [26–28]. Luciferase reporter assays were performed after transfection of c-Myc promoter-Luc construct and CTHRC1 siRNA into U87 and LN229 cells (Fig. S10A–B). We found that the knockdown of CTHRC1 significantly inhibited c-Myc transcription activity (Fig. S10B). Furthermore, the effect of HOXA11-AS and CTHRC1 on β-catenin co-activator activity was measured using the TOP/FOP luciferase reporter system, that has wild-type (TOP) or mutant (FOP) TCF binding sites upstream of a minimal c-fos promoter. TOP, but not FOP, luciferase activity was reduced after depletion of HOXA11-AS or CTHRC1 (Figs. 3C and S10C). Collectively, these results suggest that HOXA11-AS and CTHRC1 activate the β-catenin/c-Myc cascade.

We subsequently investigated whether CTHRC1 mediates HOXA11-AS activity in the β-catenin/c-Myc pathway. We performed c-Myc and TOP/FOP luciferase reporter assays by transfecting U87 and LN229 cells with Lv-HOXA11-AS and CTHRC1 siRNA, individually and together. As shown in Figs. 3D and 3E, knockdown of CTHRC1 completely abrogated HOXA11-AS stimulated c-Myc and β-catenin transcription activities, and the introduction of siRNA-CTHRC1 alone repressed the basal c-Myc promoter-Luc and TOP-Luc activity in both cell lines. Since HOXA11-AS serves as a ceRNA for let-7b-5p to induce CTHRC1, we further examined the effect of let-7b-5p on HOXA11-AS-regulated β-catenin/c-Myc pathway. As shown in Fig. 3F, the HOXA11-AS knockdown reduced c-Myc transcriptional activity and depletion of let-7b-5p largely reversed the inhibitory effect of HOXA11-AS knockdown on c-Myc transcriptional activity (Figs. 3F and S10D). Taken together, these findings indicate that HOXA11-AS can activate the β-catenin/c-Myc pathway by preventing let-7b-5p from downregulating CTHRC1 expression.

Studies have shown that c-Myc is an important transcription factor that regulates the transcription of multiple oncogenes [25]. The binding site of c-Myc was found in the HOXA11-AS promoter region in MCF-7 breast cancer cell lines from UCSC (Fig. S10E), indicating that c-Myc may bind to the promoter region of HOXA11-AS in glioma. ChIP-PCR experiments further verified that c-Myc could bind to the promoter region of HOXA11-AS, and the main binding site was in the −1500bp to −1000bp region of the HOXA11-AS promoter in U87 and LN229 cells (Fig. 3G).

These results suggest that HOXA11-AS can regulate let-7b-5p/CTHRC1/β-catenin/c-Myc pathway to mediate its own transcription and form a unique self-activation loop. These results also confirmed the molecular mechanism underlying the abnormal expression of HOXA11-AS in glioma.

### Identification of the nuclear target of HOXA11-AS using transcriptomics and proteomics analyses

**Fig. 4:**
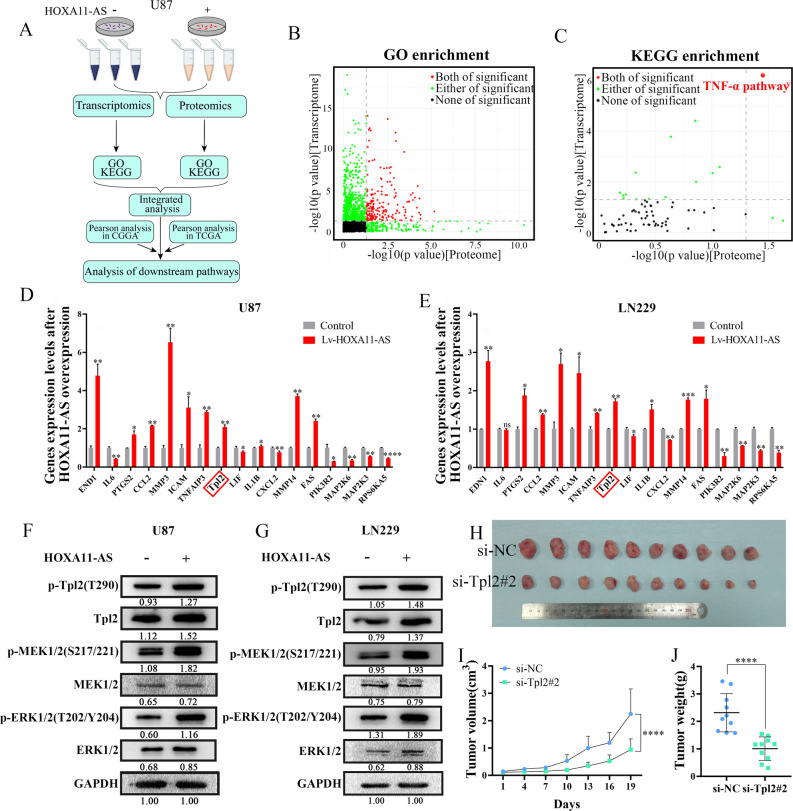
TNF-α pathway was an important downstream pathway for HOXA11-AS via integrated analysis of transcriptomics and proteomics. **A** The overall workflow of integrated analysis of transcriptomics and proteomics. **B** Scatterplot of GO items in both transcriptomic and proteomic data sets. **C** Scatterplot of KEGG items in both transcriptomic and proteomic data sets. The red plots indicated these items significant enrichment of both omics; the green plots indicated these items enrichment of single omics; the black plots indicated these items were not enriched in both omics. The expression levels of 17 DEGs enriched in the TNF-α pathway from transcriptomics were verified by RT-qPCR in U87 (**D**) and LN229 (**E**) cells. Each data point was calculated from averages of biological triplicates. Key proteins in TNF-α pathway were validated by western blot in U87 (**F**) and LN229 (**G**) cells infected with Lv-HOXA11-AS. GAPDH was used as an internal control. Xenograft mouse models were generated by subcutaneous injection of U87 cells into nude mice. Seven days after implanted, mice were treated with si-NC (*n* = 10) or si-Tpl2 (*n* = 10). Representative image of tumor dissected tumors (**H**), growth curve (**I**) and tumor weight (**J**) in si-NC or si-Tpl2-treated xenograft mouse models. *P < 0.05, **P < 0.01, ***P < 0.001, ****P < 0.0001.

As HOXA11-AS is predominantly located in the nucleus (Fig. 2A–B), we performed transcriptomics and proteomics analyses to identify its nuclear targets in U87 cells, following their transfection with Lv-HOXA11-AS or the Lv-NC control (Fig. 4A). Transcriptomics and proteomics analyses revealed that 615 genes (FC > 1.5, *P* < 0.05) and 136 proteins (FC > 1.2, *P* < 0.05) were significantly differentially expressed between U87-Lv-HOXA11-AS and U87-Lv-NC cells (Fig. S11A–B).

Next, we performed gene ontology (GO) and Kyoto Encyclopedia of Genes and Genomes (KEGG) enrichment analyses of these 615 differentially expressed genes (DEGs) and 136 differentially expressed proteins (DEPs), individually (Fig. S11C–F) and collectively (Fig. 4B–C). GO and KEGG analysis revealed that the DEGs were primarily enriched in signal transduction, regulation of signal transduction, and the TNF and TGF-β signaling pathways (Fig. S11C–D), whereas the DEPs primarily affected chromatin silencing, chromosome organization and TNF signaling pathway (Fig. S11E–F). Notably, integrated analysis of 615 DEGs and 136 DEPs revealed that, while multiple terms were common in GO enrichment analysis (Fig. 4B), only the TNF-α pathway was shared by the DEGs and DEPs in KEGG enrichment analysis (Fig. 4C), indicating that the TNF-α pathway could be an important nuclear target of HOXA11-AS. In addition, to further identify the function of HOXA11-AS involvement in glioma, Pearson correlation and cluster analysis were performed on CGGA and TCGA cohorts according to the expression pattern of HOXA11-AS. Heatmaps indicated that a large number of genes were positively or negatively correlated with HOXA11-AS in CGGA and TCGA databases (Fig. S12A–C). KEGG pathway analysis revealed that HOXA11-AS was highly correlated with TNF-α pathway in CGGA and TCGA cohorts (Fig. S12B–D), indicating that the TNF-α pathway might be an important downstream pathway for HOXA11-AS.

### HOXA11-AS regulates the malignant phenotype of glioma *via* the TNF-α pathway

Next, we performed RT-qPCR and identified 17 DEGs in the TNF-α pathway through transcriptomics analysis, 13 of which were upregulated and the others were downregulated, in Lv-HOXA11-AS infected U87 and LN229 cells (Fig. 4D–E). Among the 13 genes upregulated by HOXA11-AS, tumor progression locus 2 (Tpl2, also known as COT or MAP3K8) is a hub gene of the TNF-α pathway [29, 30].

Tpl2 regulates the occurrence and progression of tumor cells by activating the MEK/ERK pathway [31]. To investigate the role of Tpl2 and its association with HOXA11-AS in glioma, we demonstrated that the protein and phosphorylation levels of Tpl2, and activation of MEK1/2-ERK1/2, were induced by the ectopic expression of HOXA11-AS in U87 and LN229 cells (Fig. 4F–G). Furthermore, GTEx and TCGA database analyses revealed that Tpl2 expression in GBM was higher than that in LGG and normal brain tissues (Fig. S13A). Moreover, analyses of the CGGA, TCGA, REMBRANDT, and GSE16011 databases indicated that Tpl2 expression was closely correlated with the WHO grade and prognosis of glioma (Fig. S14). Correlation analysis in TCGA database and 41 glioma tissues showed that HOXA11-AS expression was positively correlated with Tpl2 expression (Fig. S13B–C). Glioma patients with high levels of both HOXA11-AS and Tpl2 expression had a poorer prognosis than those with overexpression of either alone (Fig. S13D–E).

Next, we evaluated the effect of Tpl2 on glioma growth and progression. CCK-8, wound healing, and transwell assays showed that ectopic expression of Tpl2 promoted, while knockdown of Tpl2 inhibited glioma cell proliferation, migration, and invasion (Figs. S13F–K and S15A–H). Furthermore, we established a glioma xenograft mouse model using U87 cells. We found that Tpl2 siRNA treatment significantly reduced tumor growth, weight, and size (Fig. 4H–J). Notably, the proliferation, migration, and invasion of glioma cells induced by the ectopic expression of HOXA11-AS were largely inhibited by the knockdown of Tpl2 (Fig. S16A–F). These results indicate that Tpl2 is a key mediator of HOXA11-AS and plays a pivotal role in glioma growth.

### HOXA11-AS activates Tpl2 transcription by recruiting c-Jun to Tpl2 promoter

**Fig. 5:**
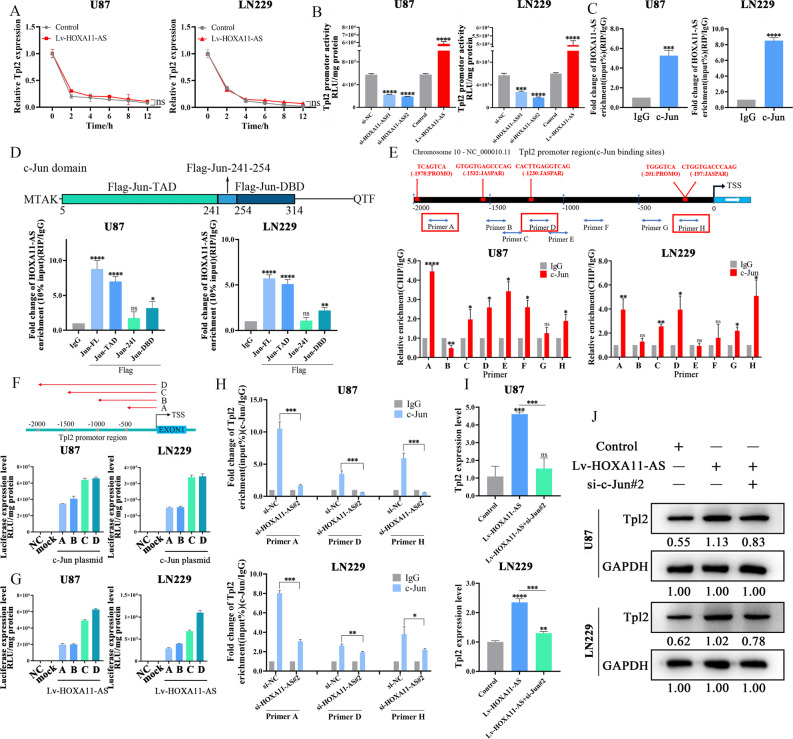
HOXA11-AS served as a scaffold to recruit c-Jun to the Tpl2 promoter in nucleus. **A** Effect of HOXA11-AS on Tpl2 mRNA stability was confirmed via ActD assay. **B** Effect of HOXA11-AS on Tpl2 transcriptional activity was verified by luciferase assay. **C** The combination of HOXA11-AS and c-Jun was verified by RIP experiment. **D** The binding of HOXA11-AS to four different domains of c-Jun (Jun-FL, Jun-TAD, Jun-241 and Jun-DBD) was verified by luciferase assay in U87 and LN229 cells. **E** The binding sites of c-Jun to the Tpl2 promoter region were verified by ChIP assay. **F** Luciferase assay was used to determine the region of the Tpl2 promoter that might interact with c-Jun. **G** Luciferase assay was used to determine the region of the Tpl2 promoter that might interact with HOXA11-AS. **H** C-Jun binding to Tpl2 promoter region depending on HOXA11-AS was determined by ChIP assay. **I**, **J** HOXA11-AS regulating Tpl2 expression depending on c-Jun was confirmed via RT-qPCR and western blot. *P < 0.05, **P < 0.01, ***P < 0.001, ****P < 0.0001.

To elucidate the mechanism by which HOXA11-AS induces Tpl2 mRNA expression, we performed an actinomycin D chase assay and found that HOXA11-AS had no effect on Tpl2 mRNA stability (Fig. 5A). However, Tpl2 promoter luciferase assay showed that the depletion of HOXA11-AS inhibited, whereas ectopic expression of HOXA11-AS stimulated, Tpl2 transcription activity (Fig. 5B).

It is well documented that lncRNAs can recruit transcription factors (TFs) to the promoter region of target genes [32]. Therefore, we hypothesized that HOXA11-AS recruits TF to the Tpl2 promoter region. PROMO database analysis revealed TFs that can bind to the Tpl2 promoter (Fig. S17A). Among these TFs, c-Jun was the most important, as it is a major downstream TF of the MEK-ERK pathway [33, 34]. Further analysis of the UCSC database revealed c-Jun binding sites within the Tpl2 promoter in A549 lung cancer cells (Fig. S17B). In addition, analysis of the Tartaglialab database (http://www.tartaglialab.com/) revealed possible binding of HOXA11-AS to c-Jun (Fig. S17C). These findings strongly suggested that HOXA11-AS might recruit c-Jun to the Tpl2 promoter.

To confirm these predictions, we performed RIP experiments in U87 and LN229 cells using a c-Jun antibody for immunoprecipitation, and found that HOXA11-AS bound to c-Jun (Fig. 5C). We then created Flag-c-Jun deletion mutants, including Jun-transactivation domain (TAD), Jun-241-254 and Jun-DNA binding domain (DBD) (See Fig. 5D for the schematic diagram of the c-Jun deletion mutants). The binding of HOXA11-AS to four different domains of c-Jun was verified by RIP assay in U87 and LN229 cells. The results showed that the TAD of c-Jun interacted with HOXA11-AS (Fig. 5D). Subsequently, ChIP assay revealed that c-Jun binds to the Tpl2 promoter, and the regions corresponding to primers A, D and H are the location where c-Jun is most likely to bind Tpl2 (Fig. 5E). To determine the region of the Tpl2 promoter that interacts with c-Jun/HOXA11-AS, we constructed luciferase plasmids with four different Tpl2 promoter regions, −500bp/0, −1000bp/0, −1500bp/0 and −2000bp/0 respectively. The luciferase reporter assay demonstrated that c-Jun/HOXA11-AS bound to the −1000 to −1500bp region in the Tpl2 promoter (Fig. 5F–G). In addition, the ChIP assay revealed that the binding of c-Jun to the Tpl2 promoter was largely abrogated by knockdown of HOXA11-AS (Fig. 5H). Furthermore, we investigated whether the induction of Tpl2 by HOXA11-AS depended on the presence of c-Jun. After infection of U87 and LN229 cells with Lv-HOXA11-AS alone and together with c-Jun siRNA, RT-qPCR and western blotting analyses showed that HOXA11-AS-induced Tpl2 mRNA and protein expression was significantly reduced by c-Jun depletion (Figs. 5I–J and S17D). Collectively, these results indicate that HOXA11-AS recruits c-Jun to the Tpl2 promoter to activate Tpl2/MEK/ERK pathway.

### HOXA11-AS knockdown increased the sensitivity of glioma cells to ROS

Cancer cells are more sensitive to the accumulation of ROS because they have higher basal levels of ROS, owing to the enhanced antioxidant capacity [35–37]. Increasing intracellular ROS levels to further aggravate oxidative stress in tumor cells can help cross the toxicity threshold in cancer cells before normal cells, and thus selectively kill cancer cells. The MEK-ERK pathway can affect the antioxidant capacity of cancer cells, including glioma [38, 39]. The above results indicate that HOXA11-AS regulates the activity of the Tpl2-MEK1/2-ERK1/2 pathway. Therefore, we hypothesized that HOXA11-AS may affect the sensitivity of glioma cells to ROS.

**Fig. 6:**
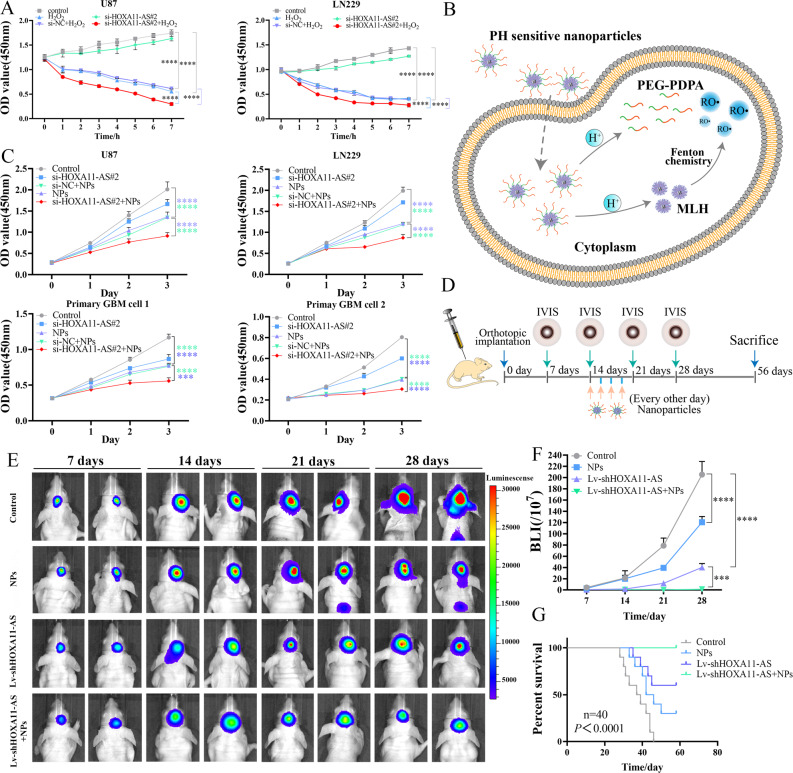
HOXA11-AS regulated glioma sensitivity to ROS in vivo and in vitro. **A** HOXA11-AS knockdown affecting the sensitivity of glioma cells to H_2_O_2_ was verified by CCK-8 assay. **B** Schematic representation of the use of MLH-carrying nanoparticles (NPs) for producing reactive oxygen species (ROS). **C** HOXA11-AS knockdown affecting the sensitivity of glioma and patient-derived primary GBM cells to ROS produced by NPs was determined via CCK-8 assay in vitro. **D** The scheme of tumor inoculation and systemic injection. Bioluminescent images (**E**) and bioluminescent intensity (**F**) of the glioma-bearing mice 7, 14, 21, and 28 days after treatment with NPs, Lv-shHOXA11-AS or cotreatment with NPs and Lv-shHOXA11-AS. **G** Kaplan-Meier survival analysis of mice bearing orthotopically transplanted U87 cells. Group 1: treated with PBS and Lv-shNC (control). Group 2: treated with NPs alone (13.33 μmol/kg, i.p. injection). Group 3: treated with Lv-shHOXA11-AS alone (Intracranial implantation of U87 cells infected with HOXA11-AS shRNA lentivirus). Group 4: cotreated with Lv-shHOXA11-AS and NPs (Intracranial implantation of U87 cells transfected with Lv-shHOXA11-AS lentivirus and 13.33μmol/kg NPs, i.p. injection). ***P < 0.001, ****P < 0.0001.

To test this hypothesis, we first verified whether HOXA11-AS expression could affect ROS levels in glioma, and found that of HOXA11-AS knockdown did not affect intracellular ROS levels in U87 and LN229 cells (Fig. S17E). Then, we used hydrogen peroxide(H_2_O_2_) as a model drug to verify whether HOXA11-AS affects the sensitivity of glioma cells to ROS. The IC50 of H_2_O_2_ was found to be 27.55 to 30.27 nM in U87 cells, and 10.21 to 10.98 nM in LN229 cells, through CCK-8 assay (Fig. S18A). HOXA11-AS knockdown was found to increase the sensitivity of glioma cells to H_2_O_2_, while HOXA11-AS overexpression reduced it (Figs. 6A and S18B). For future translational research, we developed a nanoparticle (NP) platform that could promote ROS production in cancer cells. This NPs could exist stably and decompose into PEG-PDPA and MLH under the action of hydrogen ions after entering cells. Iron ions (labile iron pool, LIP) could react with MLH and promote the decomposition of MLH into RO· (peroxide). The peroxide compounds released from NPs produce intracellular ROS and kill the tumor cells (Fig. 6B) [21]. The particle size of NPs was about 50 nm verified by DLS experiment (Fig. S18C). Two GBM cell lines and two patient-derived primary GBM cells were used to study the role of HOXA11-AS in increasing the resistance of cancer cells to ROS. In vitro ROS sensitization experiments showed that HOXA11-AS knockdown increased the sensitivity of glioma cells to ROS produced by NPs, and vice versa. (Figs. 6C and S18D–G). These results indicated that HOXA11-AS could regulate the sensitivity of glioma cells to ROS released from NPs in vitro.

To further verify HOXA11-AS could regulate the sensitivity of glioma cells to ROS released from NPs in vivo, the ability of NPs to cross the blood-brain barrier (BBB) was verified. We inoculated U87-GFP-luc cells into the brain of Balb/c nude mice to form an orthotopic glioma model, and injected Free-PPa and PPa-NPs into mice through the tail vein. Fig. S19A showed that PPa-NPs could cross the BBB compared with Free-PPa. In vivo distribution experiments showed that Free-PPa and PPa-NPs were mainly enriched in the liver and kidney (Fig. S19B). Furthermore, these PPa-NPs were able to accumulate at tumor sites (Fig. 19 C) rather than normal brain tissue (Fig. 19D) or neurons (Fig. 19E)

To investigate the ability of HOXA11-AS to regulate ROS sensitivity in vivo, xenograft tumors were established through the injection of U87 cells stably expressing bioluminescent reporter luciferase or HOXA11-AS shRNA, into the forebrain striatum and treated with NPs. The results showed that both NP treatment and HOXA11-AS knockdown alone modestly improved survival and reduced tumor burden (Fig. 6D–E). However, the combination of NP treatment and HOXA11-AS knockdown significantly reduced the tumor burden and improved survival (Fig. 6F–G). The median survival results showed that the co-treatment with NPs and HOXA11-AS knockdown group (all ten nude mice were alive at the end of the experiment on day 58) lived significantly longer than the control (38 days), NP treatment (44 days), and HOXA11-AS knockdown (six nude mice were still alive at the end of the experiment on day 58) groups (Fig. 6G). In addition, IHC analysis showed that the combination of NP treatment and HOXA11-AS knockdown significantly reduced the signals of β-catenin in nuclear, Tpl2, p-MEK and p-ERK compared with control or NPs groups (Fig. S20). Histological analysis of the mice did not reveal any deleterious effects of cotreatment of HOXA11-AS knockdown and NP in the main organs, including the heart, liver, spleen, lung, and kidneys (Fig. S21). Notably, we did not observe any physical or behavioral differences between the treatment groups and the control group. Specifically, there were no statistically significant differences in constitutional signs, including animal weights, in any of the treatment groups compared to the control group. In summary, HOXA11-AS knockdown sensitized GBM cells to ROS in xenograft models, drastically impairing tumor growth and prolonging survival.

## Discussion

Glioma is the most common malignant tumor of the central nervous system, accounting for 80% of all primary brain tumors. Despite advances in understanding the molecular and cellular biology of glioma, there have been no significant changes in treatment strategies [2]. Increasing evidence shows that many long noncoding RNAs (lncRNAs) in glioma are closely associated with tumorigenesis and prognosis [40].

HOXA11-AS is located on chromosome 7p15.2, and the length of the HOXA11-AS gene is 3,885 bp, whereas that of the HOXA11-AS transcript is 1628 nt [18]. It has been reported that HOXA11-AS could act as either an oncogene or tumor suppressor gene in many types of tumors. For example, HOXA11-AS can act as an oncogene in non-small cell lung cancer (NSCLC), hepatocellular carcinoma (HCC), glioma, breast cancer (BC), gastric cancer (GC), kidney cancer (RC), uveal melanoma (UM), laryngeal squamous cell carcinoma (LSCC), cervical cancer (CC), esophageal squamous cell carcinoma (ESCC), and osteosarcoma [16, 41–47]. In contrast, HOXA11-AS exerts a tumor suppressor effect in epithelial ovarian cancer (EOC) [48]. Our previous studies showed that oncogenic HOXA11-AS regulates glioma cell cycle progression and tumor cell stemness[16]. Based on bioinformatics analyses of glioma databases, we found that high expression of HOXA11-AS was significantly correlated with high-grade and poor prognosis in glioma. CCK-8, EdU, wound healing, and Transwell assays showed that HOXA11-AS promoted the proliferation, migration, and invasion of glioma cells. Subcutaneous xenograft experiments also verified the role of HOXA11-AS in promoting glioma growth. These results indicated that HOXA11-AS is highly expressed, and promotes the growth and progression of glioma, confirming its oncogenic function.

Subcellular location assay has shown that HOXA11-AS is mainly located in the nuclear region and a small part is located in the cytoplasm. Most lncRNAs located in the cytoplasm likely function as ceRNAs. For example, Zhan et al. found that HOXA11-AS could act as a ceRNA by absorbing miR-214-3p in HCC, while miR-214-3p could directly inhibit enhancer of zeste homolog 2 (EZH2) transcription [49]. In glioma, HOXA11-AS could regulate the expression of EZH2 by sponging miR-214-3p [50]. Since HOXA11-AS could bind to Ago2 via RIP assay, it was speculated that HOXA11-AS could function as a ceRNA. Furthermore, eight target miRNAs that might be regulated by HOXA11-AS were identified using small RNA sequencing. Through luciferase reporter assay, let-7b-5p was identified as the direct target of HOXA11-AS, and CTHRC1, the downstream target of let-7b-5p, was also discovered. which indicated that CTHRC1 is a direct target of HOXA11-AS/let-7b-5p. We further found that HOXA11-AS could regulate the β-catenin/c-Myc pathway through the let-7b-5p /CTHRC1 axis, and c-Myc could bind to the HOXA11-AS promoter. These results indicate the presence of the self-activation loop, which also explains the molecular mechanism underlying the abnormal expression of HOXA11-AS in glioma.

Since HOXA11-AS is mainly located in the nucleus of glioma cell, we continued to explore the main mechanism of HOXA11-AS in the nucleus. Studies have reported that lncRNAs located in the nucleus may function as molecular scaffolds, recruiting transcription factors to target gene promoters and thus promoting the transcription of target genes. For example, Chen et al. found that HOXA11-AS could interact with DNA (Cytosine-5)-methyltransferase 1 (DNMT1) and enhancer of zeste homolog 2 (EZH2), and recruit DNMT1 and EZH2 to the promoter region of miR-200b, thereby regulating the transcriptional activity of miR-200b. Integrated analysis of transcriptomics and proteomics showed that TNF-α pathway might be an important downstream pathway for HOXA11-AS. The key node gene Tpl2 in this pathway has attracted our attention. Through ChIP, RIP, and rescue experiments, we found that HOXA11-AS promoted Tpl2 transcription by recruiting c-Jun to its promoter, thus activating the Tpl2-MEK1/2-ERK1/2 pathway. Moreover, HOXA11-AS regulates the proliferation, migration, and invasion of glioma cells by activating the Tpl2-MEK1/2-ERK1/2 pathway.

**Fig. 7:**
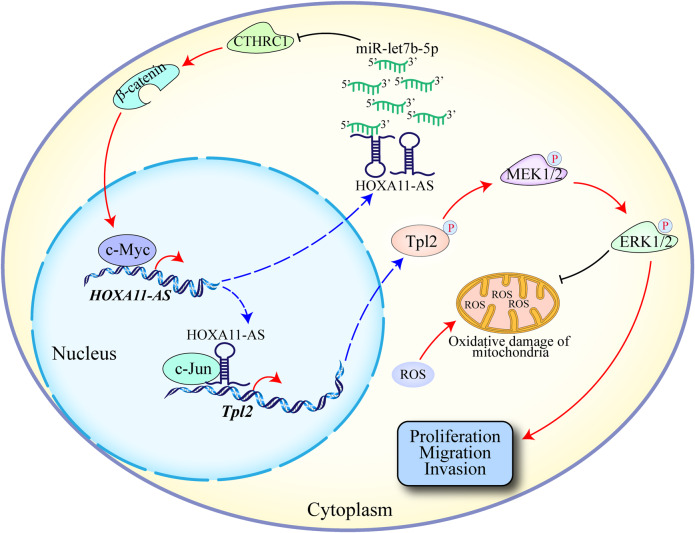
The main mechanism diagram of this study. A schematic illustrated the abnormal expression of HOXA11-AS in glioma and the mechanism of HOXA11-AS regulating the sensitivity of glioma cells to ROS.

By increasing intracellular ROS levels and inhibiting the antioxidant capacity of cancer cells to further aggravate oxidative stress, cancer cells can reach the toxicity threshold before normal cells, leading to their selective destruction. The MEK-ERK pathway can affect the antioxidant capacity of cancer cells [37]. Therefore, we speculated that HOXA11-AS might affect the sensitivity of glioma cells to ROS by regulating the Tpl2-MEK1/2-ERK1/2 pathway. Through ROS experiments, we found that HOXA11-AS increased the tolerance of glioma cells and primary GBM cells to ROS through the Tpl2-MEK1/2-ERK1/2 pathway. In vivo experiments, HOXA11-AS knockdown combined with adjuvant therapy using ROS-producing nanoparticles significantly increased the sensitivity of glioma cells to ROS. These results suggest that HOXA11-AS regulates the sensitivity of glioma cells to ROS through the Tpl2-MEK1/2-ERK1/2 pathway. Figure 7 shows the mechanism of the core pathway delineated the entire manuscript.

## Conclusion

Taken together, we found that HOXA11-AS could play an oncogenic role in glioma and is associated with poor prognosis. Cytoplasmic HOXA11-AS could upregulate the expression of CTHRC1 by adsorbing of let-7b-5p, thus activating β-catenin/c-Myc to form a self-activation loop, which may be the reason for the abnormal expression of HOXA11-AS. In addition, we also confirmed that nuclear HOXA11-AS promoted Tpl2 transcription by recruiting c-Jun to the promoter region of Tpl2, thereby activating the Tpl2-MEK1/2-ERK1/2 pathway, affecting the sensitivity of glioma to ROS. Our data suggest that HOXA11-AS can act as a promising prognostic biomarker and new therapeutical target to mediate ROS sensitivity in glioma.

## Supplementary information

A reproducibility checklist

Supplementary materials(Figure S1-S21)

Supplementary Table 1. Clinical data of glioma patients.

Supplementary Table 2. The sense and antisense siRNA sequences for mRNA or lncRNA knockdown.

Supplementary Table 3. The mimics and inhibitor sequences for let-7b-5p overexpression or knockdown.

Supplementary Table 4. The forward and reverse primers for qRT-PCR.

Supplementary Table 5. Antibody informations.

Supplementary Table 6. The odd and even probe sequences for ChIRP assay.

Supplementary Table 7. The forward and reverse primer sequences for ChIP assay.

Original full length western blots

## Data Availability

Data available on request from the authors upon reasonable request.
