# Supplementary material for: LncRNA HOXA11-AS promotes glioma malignant phenotypes and reduces its sensitivity to ROS via Tpl2-MEK1/2-ERK1/2 pathway
Source: Cell Death Dis. 2022 Nov 9;13(11):942. doi: 10.1038/s41419-022-05393-5 (PMC9646708; doi:10.1038/s41419-022-05393-5)
Supplement: Supplementary file 2 — Supplementary materials(Figure S1-S21) [file 41419_2022_5393_MOESM2_ESM.docx]

**Supplementary materials**


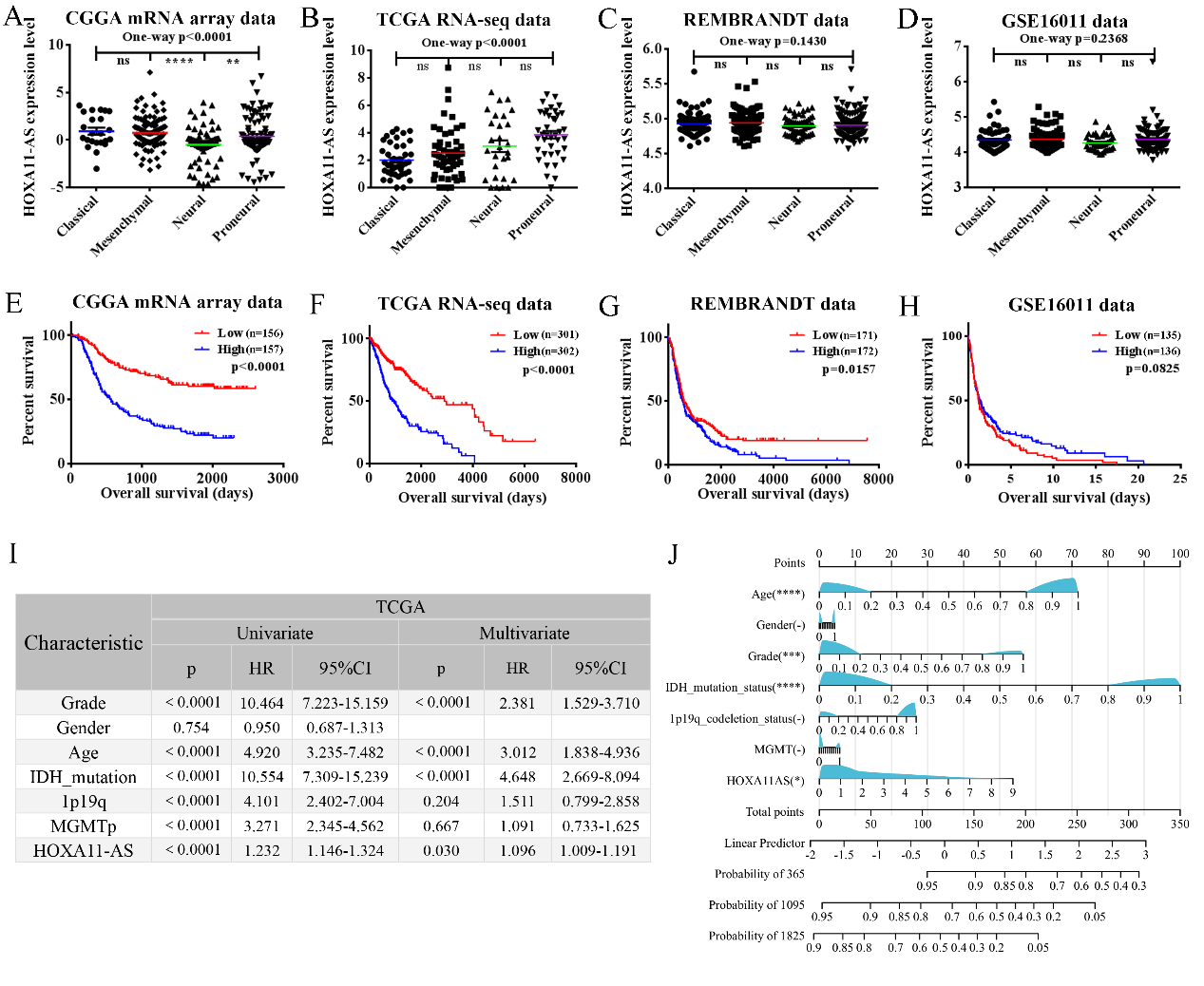


**Figure S1. Correlation of HOXA11-AS with subtypes and prognosis of glioma patients.**

Correlation of HOXA11-AS expression and subtypes of glioma patients in the CGGA **(A)**, TCGA **(B)**, REMBRANDT **(C)** and GSE16011 **(D)** datasets. Kaplan-Meier survival curves were used to examine the relationships between HOXA11-AS expression and the survival of glioma patients in the CGGA **(E)**, TCGA **(F)**, REMBRANDT **(G)** and GSE16011 **(H)** datasets. **(I)** Univariate and multivariate Cox analyses of HOXA11-AS and clinical features of glioma in TCGA database. **(J)** The nomogram model of HOXA11-AS and clinical features in TCGA dataset. **P*<0.05, ***P*<0.01, ****P*<0.001, *****P*<0.0001.

**
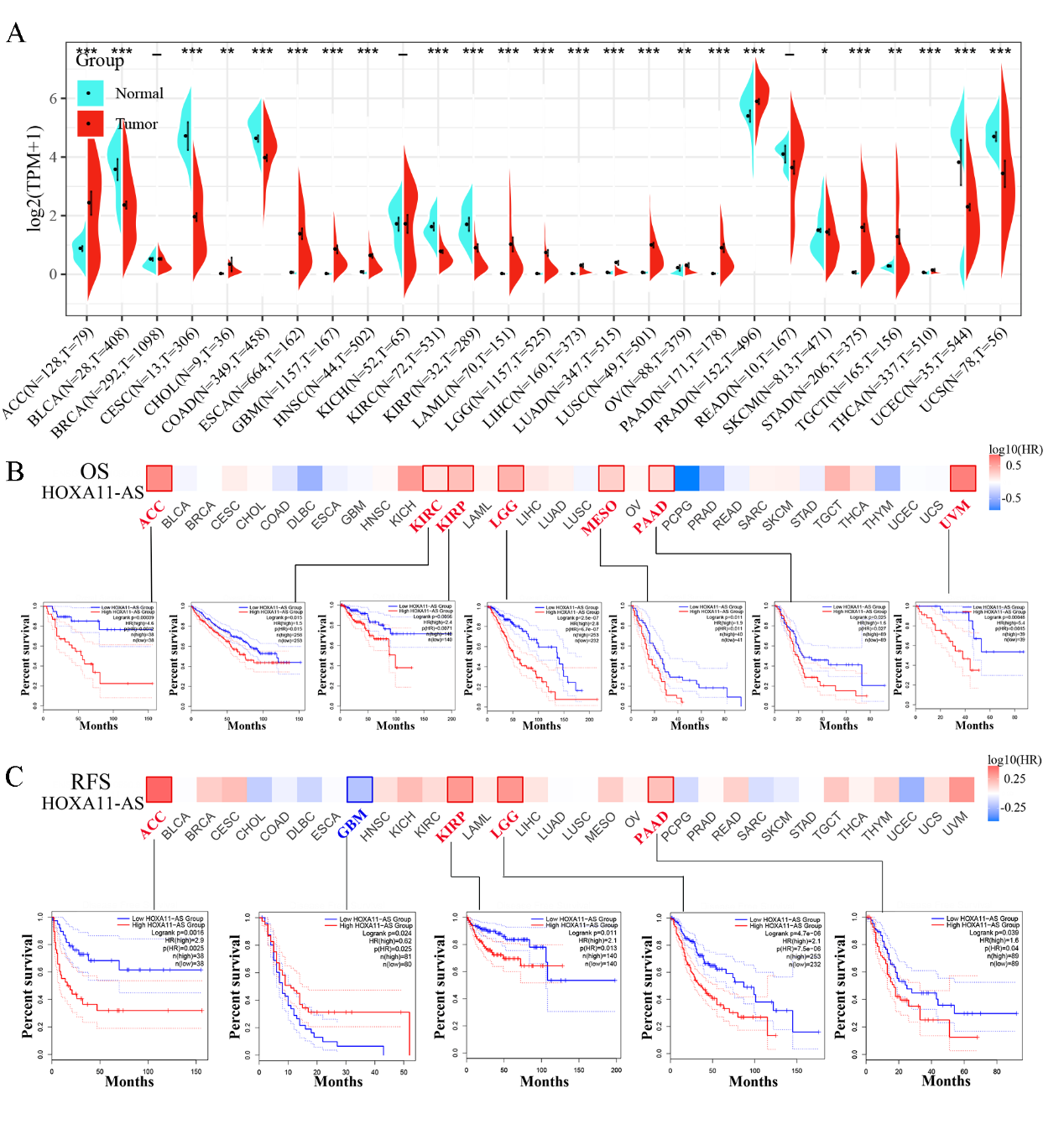
**

**Figure S2.** **The correlation between HOXA11-AS expression and prognosis in multiple cancers.**

**(A)** HOXA11-AS expression levels in different tumors and corresponding normal tissues from TCGA and GTEx databases by SangerBox. **(B)** Kaplan-Meier survival curves were used to examine the relationship between expression of HOXA11-AS and the overall survival (OS) of patients with various tumors in the TCGA cohort. **(C)** Kaplan-Meier survival curves were used to examine the relationship between expression of HOXA11-AS and the recurrence-free survival (RFS) of patients with various tumors in the TCGA cohort. **P*<0.05, ***P*<0.01, ****P*<0.001.


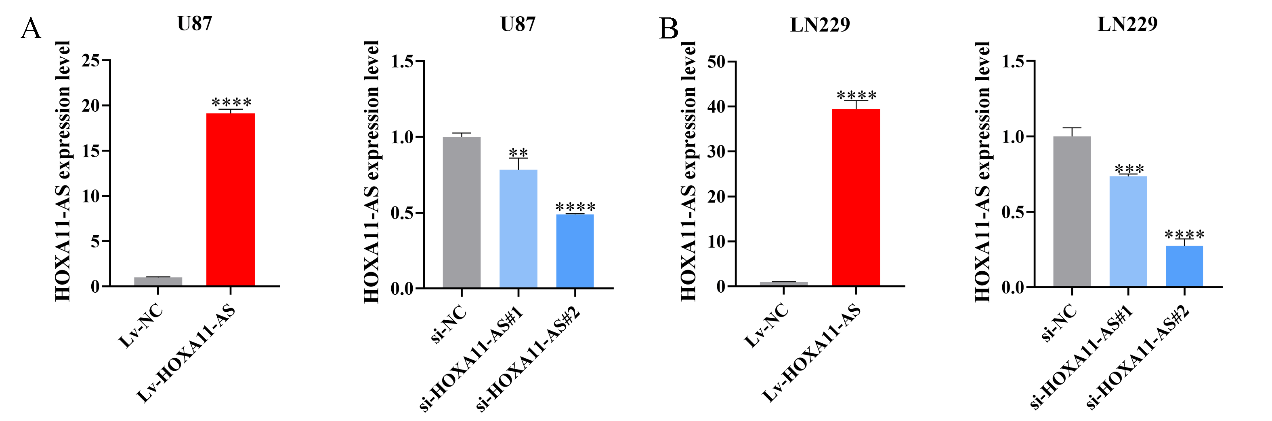


**Figure S3. Knockdown and overexpression efficiencies of HOXA11-AS** **in U87and LN229 cells.**

Knockdown and overexpression efficiencies of HOXA11-AS were verified by RT-qPCR in U87 **(A)** and LN229 **(B)** cells treated with HOXA11-AS siRNAs or Lv-HOXA11-AS. ***P*<0.01, ****P*<0.001, *****P*<0.0001.


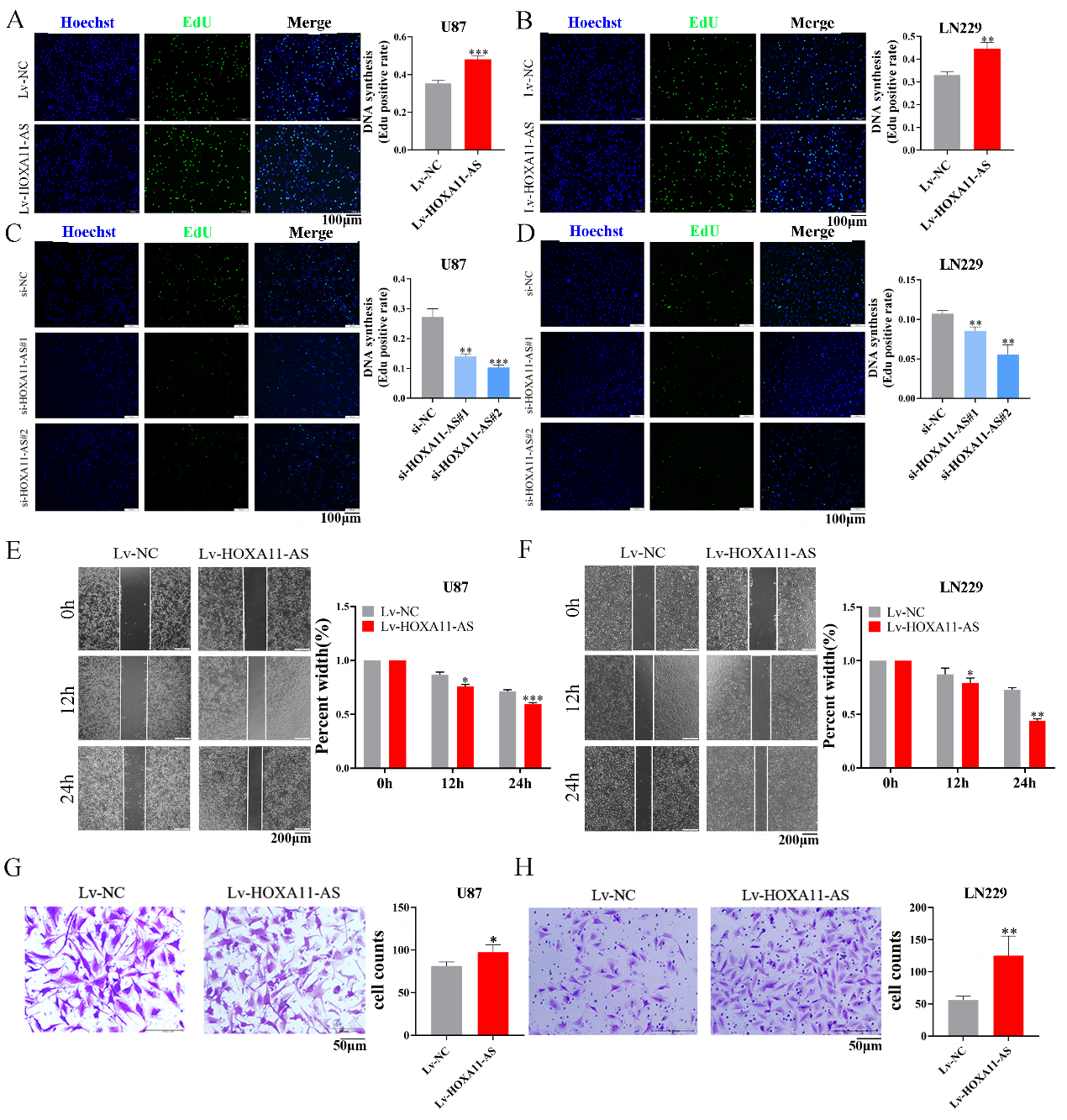


**Figure S4. HOXA11-AS promoted the proliferation, migration and invasion of glioma cells.**

The effect of HOXA11-AS overexpression on glioma proliferation was verified via EdU experiment in U87 **(A)** and LN229 **(B)** cells. The effect of HOXA11-AS knockdown on glioma proliferation was verified via EdU experiment in U87 **(C)** and LN229 **(D)** cells. The effect of HOXA11-AS overexpression on the migration was verified via wound healing assay in U87 **(E)** and LN229 cells **(F)**. The effect of HOXA11-AS overexpression on the invasion was verified via Transwell assay in U87 **(G)** and LN229 cells **(H)**. **P*<0.05, ***P*<0.01, ****P*<0.001.


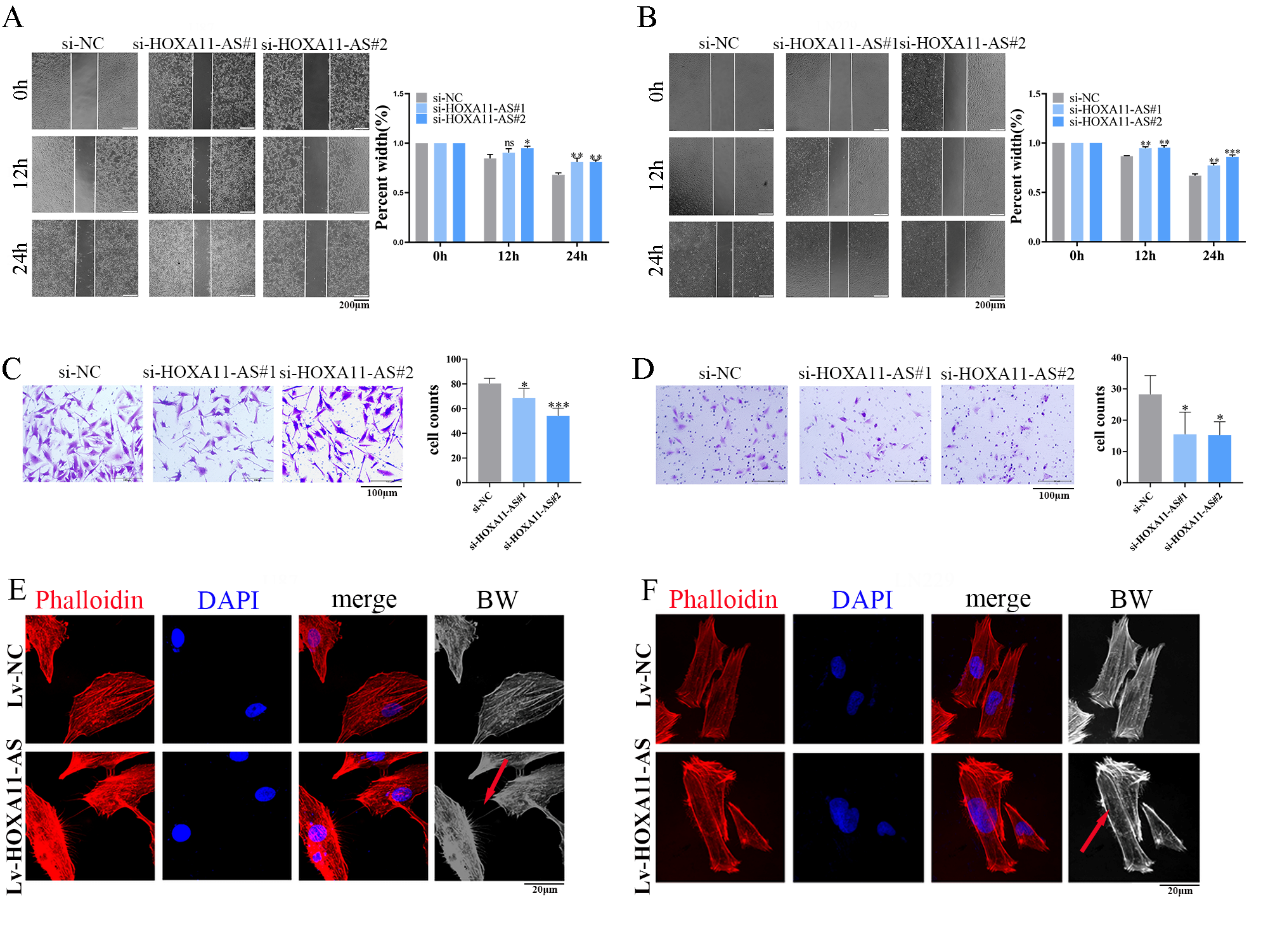


**Figure S5**. **HOXA11-AS promoted the migration and invasion of glioma cells.**

The effect of HOXA11-AS knockdown on the migration was verified via wound healing assay in U87 **(A)** and LN229 cells **(B)**. The effect of HOXA11-AS knockdown on the invasion was verified via Transwell assay in U87 **(C)** and LN229 cells **(D)**. The effect of HOXA11-AS overexpression on glioma cell migration was verified via phalloidin staining assay in U87 **(E)** and LN229 cells **(F)**. **P*<0.05, ***P*<0.01, ****P*<0.001.

**
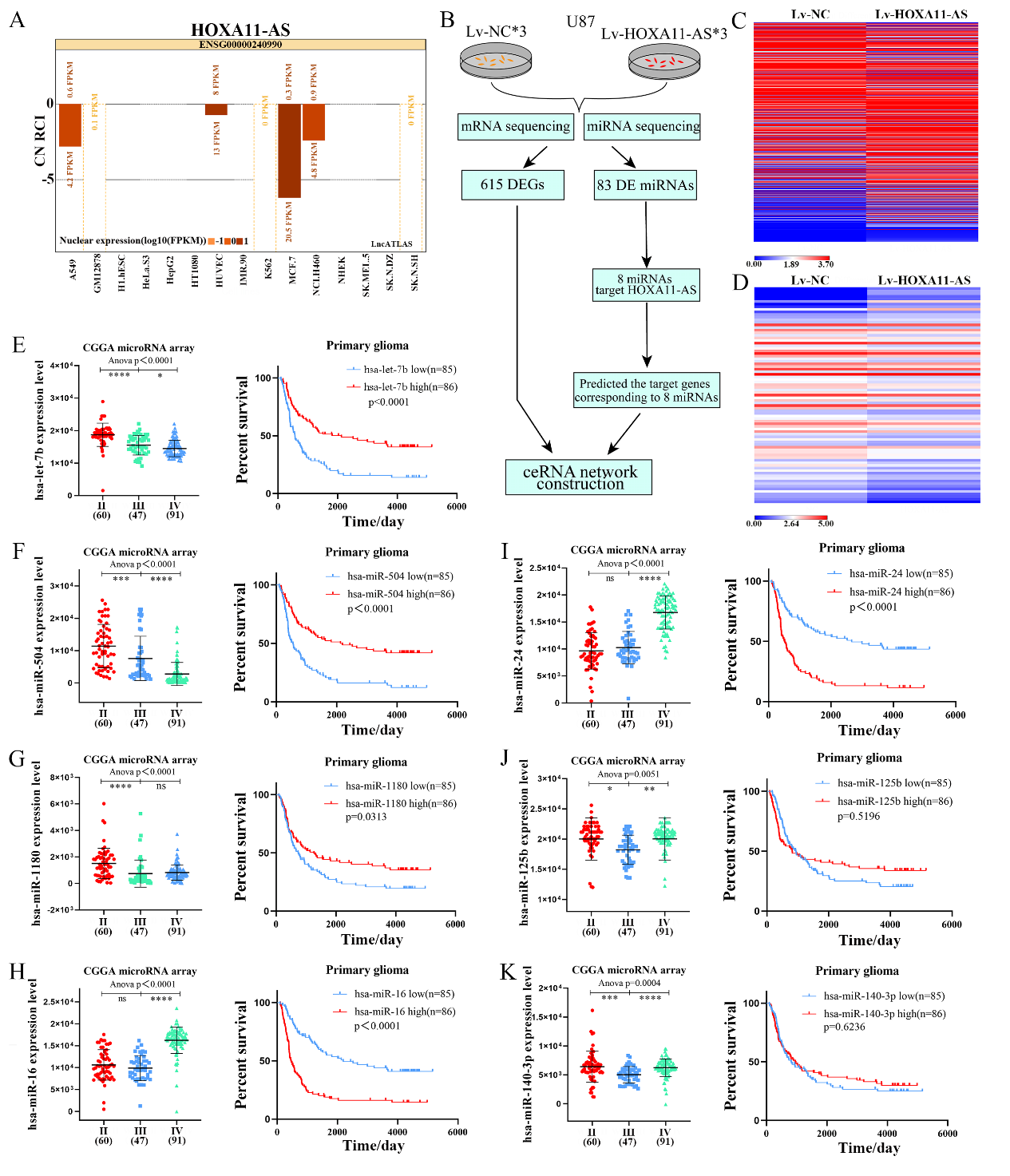
**

**Figure S6.** **The downstream miRNA targets of HOXA11-AS were analyzed by mRNA and small RNA sequencing.**

**(A)** The subcellular localization of HOXA11-AS in different tumor cell lines was analyzed in lncATLAS database. **(B)** The overall workflow of mRNA and small RNA sequencing after overexpression of HOXA11-AS in U87 cell line. **(C)** Heat map of 615 differentially expressed genes (DEGs) (|FC| > 1.5, *P* < 0.05) between Lv-NC and Lv-HOXA11-AS-infected U87 cells, with rows representing mRNAs and columns representing Lv-NC and Lv-HOXA11-AS-infected U87 cells. **(D)** Heat map of 83 differentially expressed miRNAs (DE-miRNAs) (|FC| > 1.5, *P* < 0.05) between Lv-NC and Lv-HOXA11-AS-infected U87 cells, with rows representing miRNAs and columns representing Lv-NC and Lv-HOXA11-AS-infected U87 cells. **(E-K)** The relationship between miRNA expression and grade or prognosis of glioma patients was analyzed in the CGGA database. **P*<0.05, ***P*<0.01, ****P*<0.001, *****P*<0.0001.


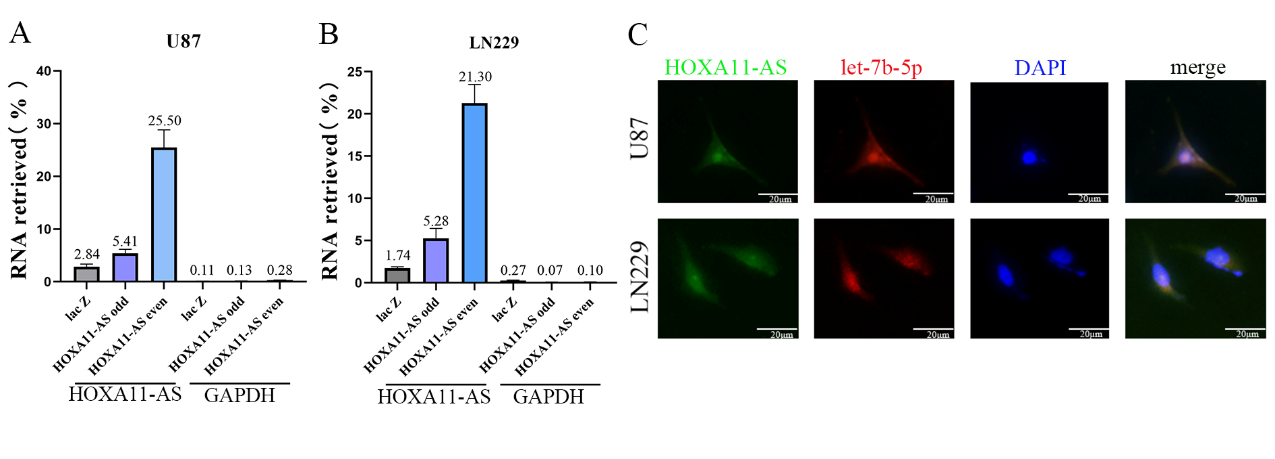


**Figure S7. HOXA11-AS could interact with let-7b-5p.**

**(A-B)** The retrieved efficiency of HOXA11-AS probe in ChIRP experiment was verified by RT-qPCR in U87 and LN229 cells. **(C)** The co-localization of HOXA11-AS and let-7b-5p in glioma cells was confirmed by Immunofluorescence assay.


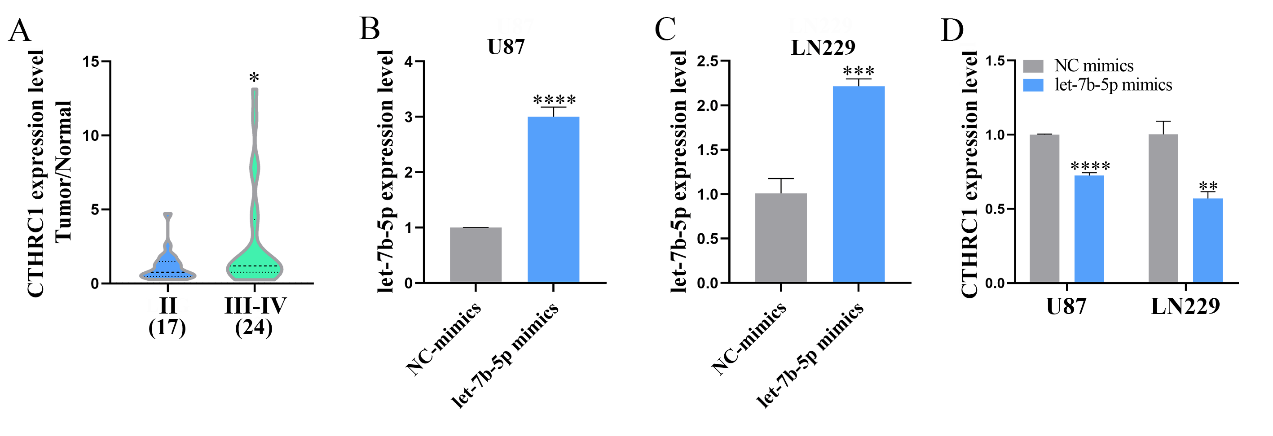


**Figure S8. Expression level of CTHRC1 after let-7b-5p mimics transfected.**

**(A)** The expression of CTHRC1 mRNA was analyzed in 41 glioma samples, including WHO II (n=17) and WHO III-IV (n=24). **(B-C)** Let-7b-5p expression level in glioma cells transfected with let-7b-5p mimics was verified by RT-qPCR. **(D)** The expression level of CTHRC1 mRNA in glioma cells transfected with let-7b-5p mimics was determined via RT-qPCR. ***P*<0.01, ****P*<0.001, *****P*<0.0001.


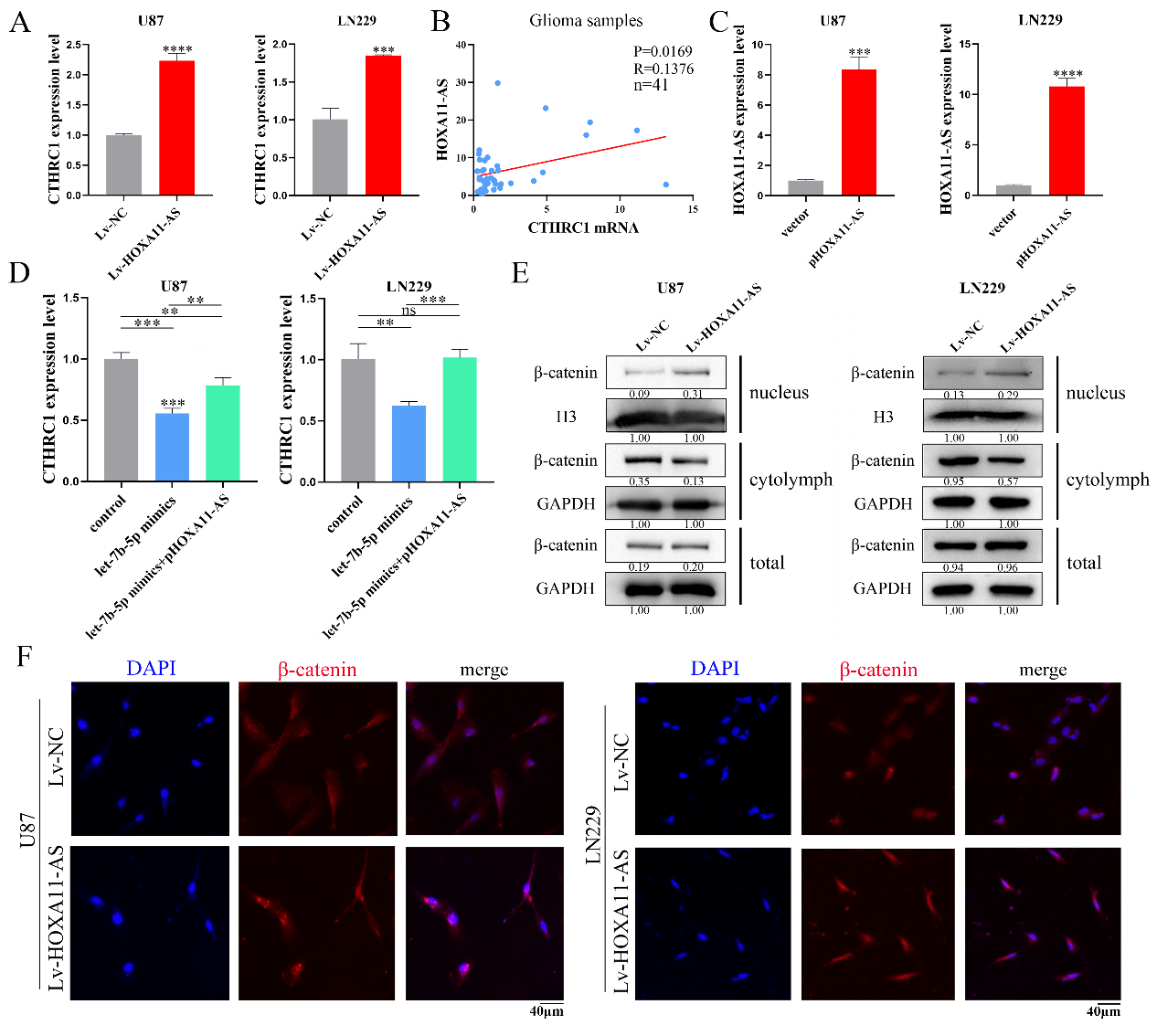


**Figure S9.** **HOXA11-AS regulated subcellular localization of β-catenin.**

**(A)** The effect of HOXA11-AS overexpression on the expression level of CTHRC1 mRNA was verified by RT-qPCR. **(B)** The correlation between CTHRC1 mRNA expression level and HOXA11-AS expression level in 41 glioma samples was analyzed by RT-qPCR. **(C)** The expression level of HOXA11-AS after U87 and LN229 cells transfected with HOXA11-AS plasmid (pHOXA11-AS). **(D)** HOXA11-AS regulating CTHRC1 mRNA expression depending on let-7b-5p was ascertained by RT-qPCR. The changes of β-catenin subcellular localization after HOXA11-AS overexpression were confirmed by western blot **(E)** and immunofluorescence assay **(F)**. ***P*<0.01, ****P*<0.001, *****P*<0.0001.


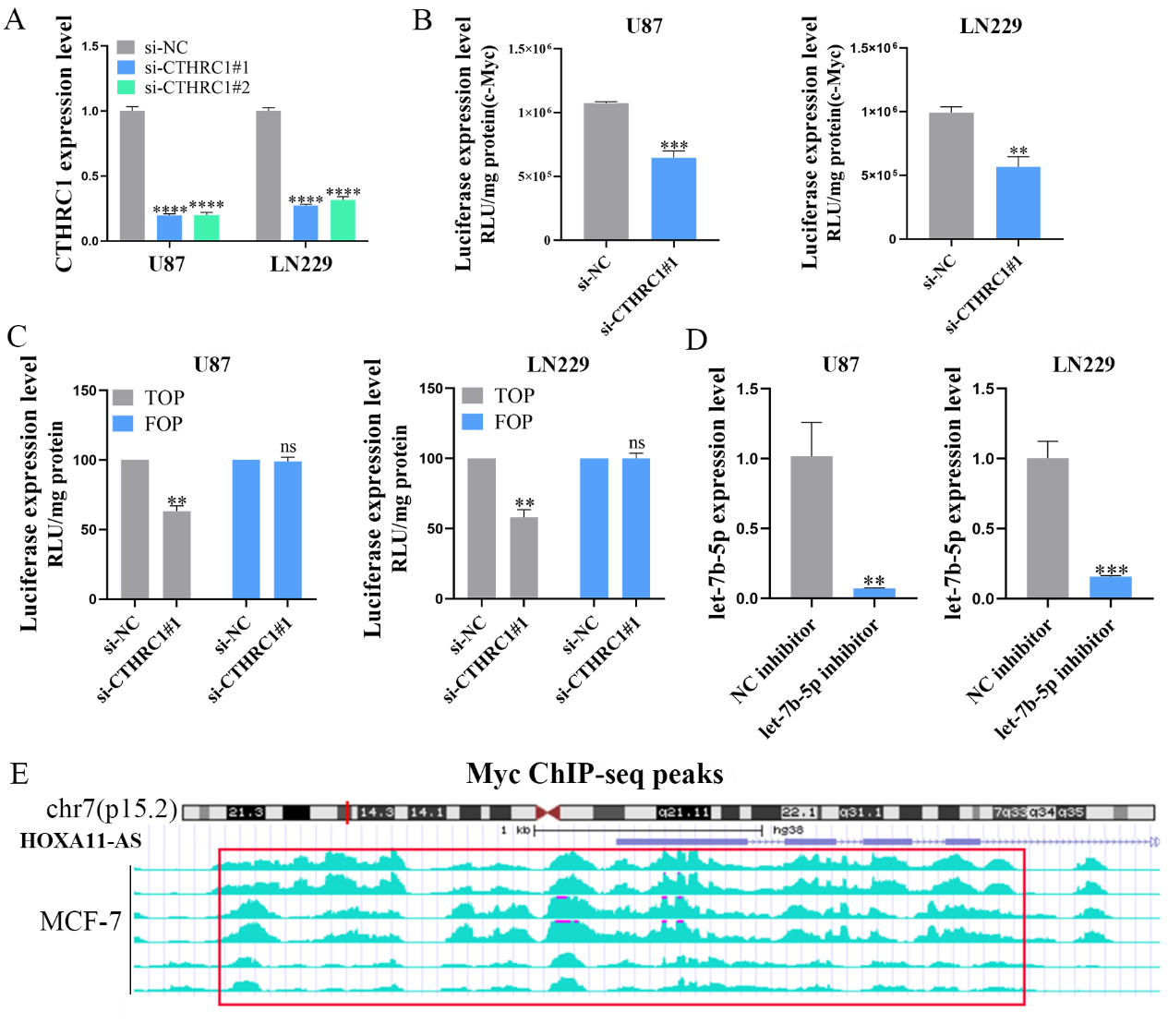


**Figure S10.** **CTHRC1 regulated the transcriptional activities of β-catenin and c-Myc.**

**(A)** The expression of CTHRC1 in U87 and LN229 cells transfected with CTHRC1 siRNAs. **(B)** The effect of CTHRC1 knockdown on c-Myc transcriptional activity was verified by luciferase assay. **(C)** The effect of CTHRC1 knockdown on β-catenin transcriptional activity was verified by TOP/FOP assay. **(D)** The expression level of let-7b-5p in U87 and LN229 cells transfected with let-7b-5p inhibitor. **(E)** The c-Myc binding peaks in the HOXA11-AS promoter region of MCF-7 cells were detected in UCSC database. ***P*<0.01, ****P*<0.001, *****P*<0.0001.

**
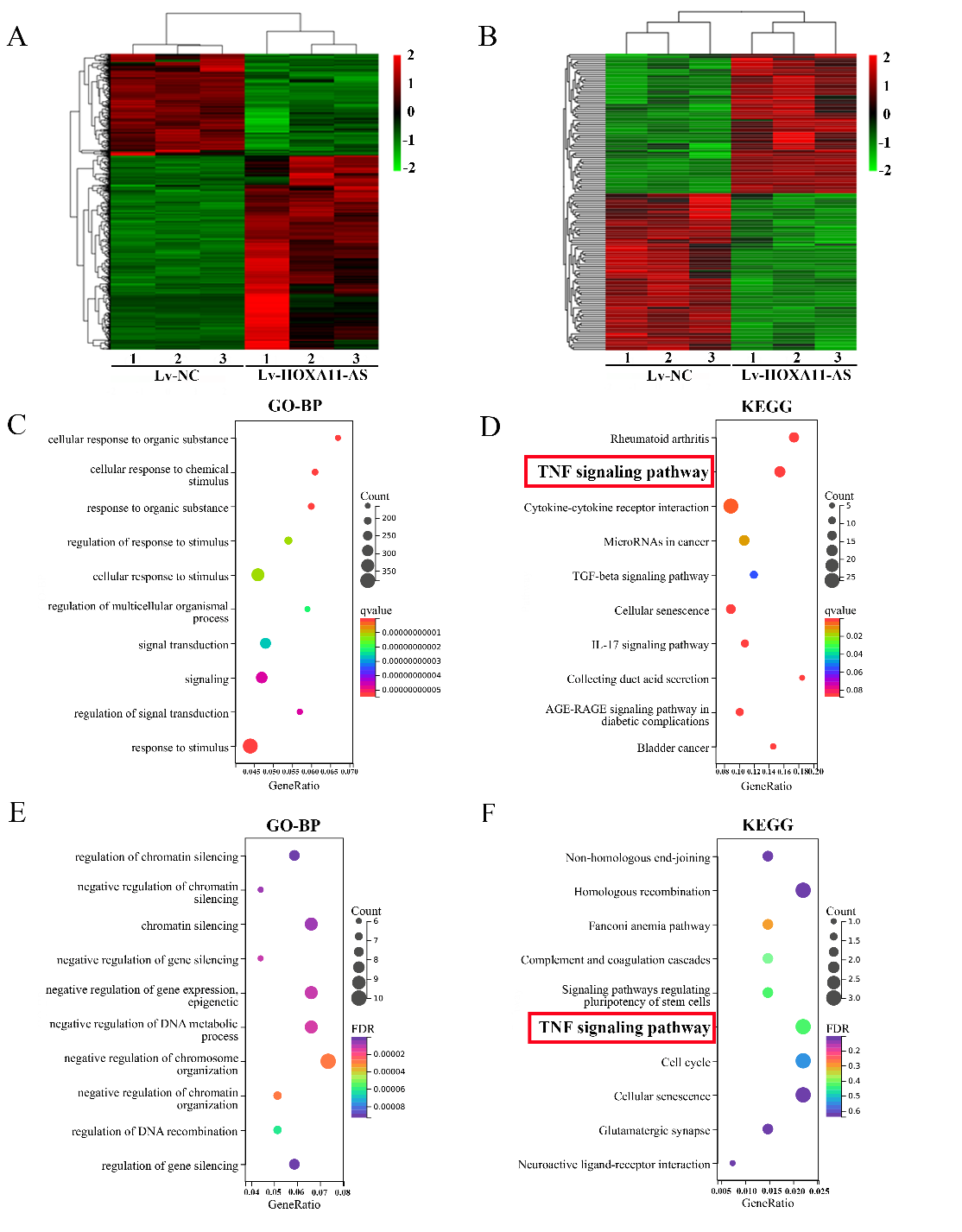
**

**Figure S11. GO-BP and KEGG analyses of transcriptomics and proteomics.**

**(A)** Heat map of 615 differentially expressed genes (DEGs) between control and Lv-HOXA11-AS- infected U87 cells was displayed, with rows representing mRNAs and columns representing glioma cells infected with Lv-NC and Lv-HOXA11-AS. **(B)** Heat map of 136 differentially expressed proteins (DEPs) between control and Lv-HOXA11-AS- infected U87 cells, with rows representing proteins and columns representing glioma cells infected with Lv-NC and Lv-HOXA11-AS. GO-BP **(C)** and KEGG **(D)** enrichment analysis of 615 DEGs in transcriptomic data was displayed. GO-BP **(E)** and KEGG **(F)** enrichment analysis of 136 DEPs in proteomic data was displayed.


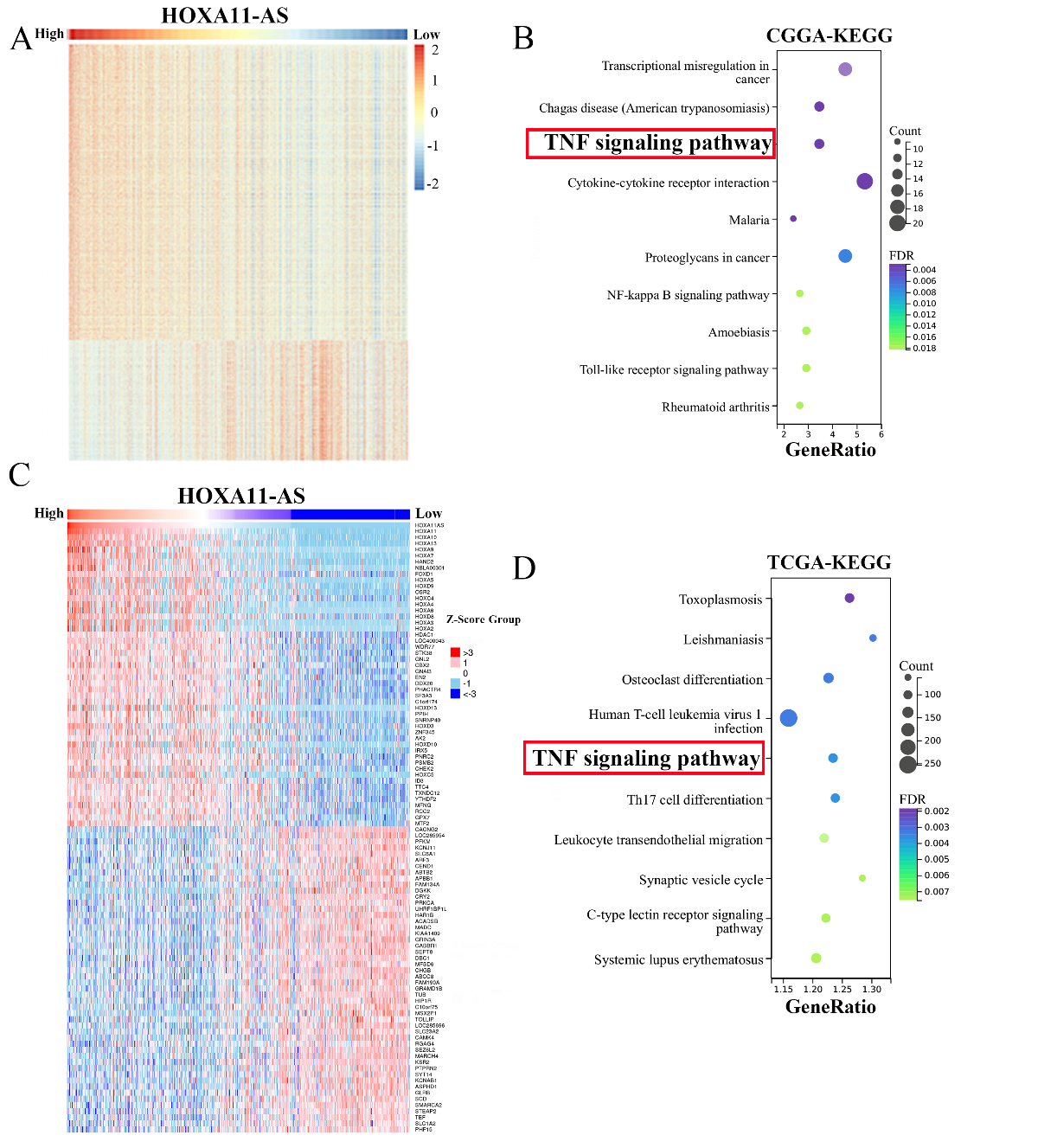


**Figure S12. KEGG analysis of HOXA11-AS associated genes in CGGA and TCGA databases.**

**(A)** Correlation analysis was performed on 693 samples with RNA-seq from the CGGA cohort. A heatmap of the relative expression levels of HOXA11-AS-associated genes in glioma tissues sorted by the level of HOXA11-AS expression was shown (|r| > 0.5, *P* < 0.05). **(B)** KEGG pathway was analyzed using the HOXA11-AS-associated genes in CGGA database. **(C)** Heatmap of the relative expression levels of HOXA11-AS positively associated genes (Top 50) and negatively associated genes (Top 50) in glioma tissues sorted by the level of HOXA11-AS expression in TCGA dataset. **(D)** KEGG pathway was analyzed using the HOXA11-AS-associated genes in TCGA database.


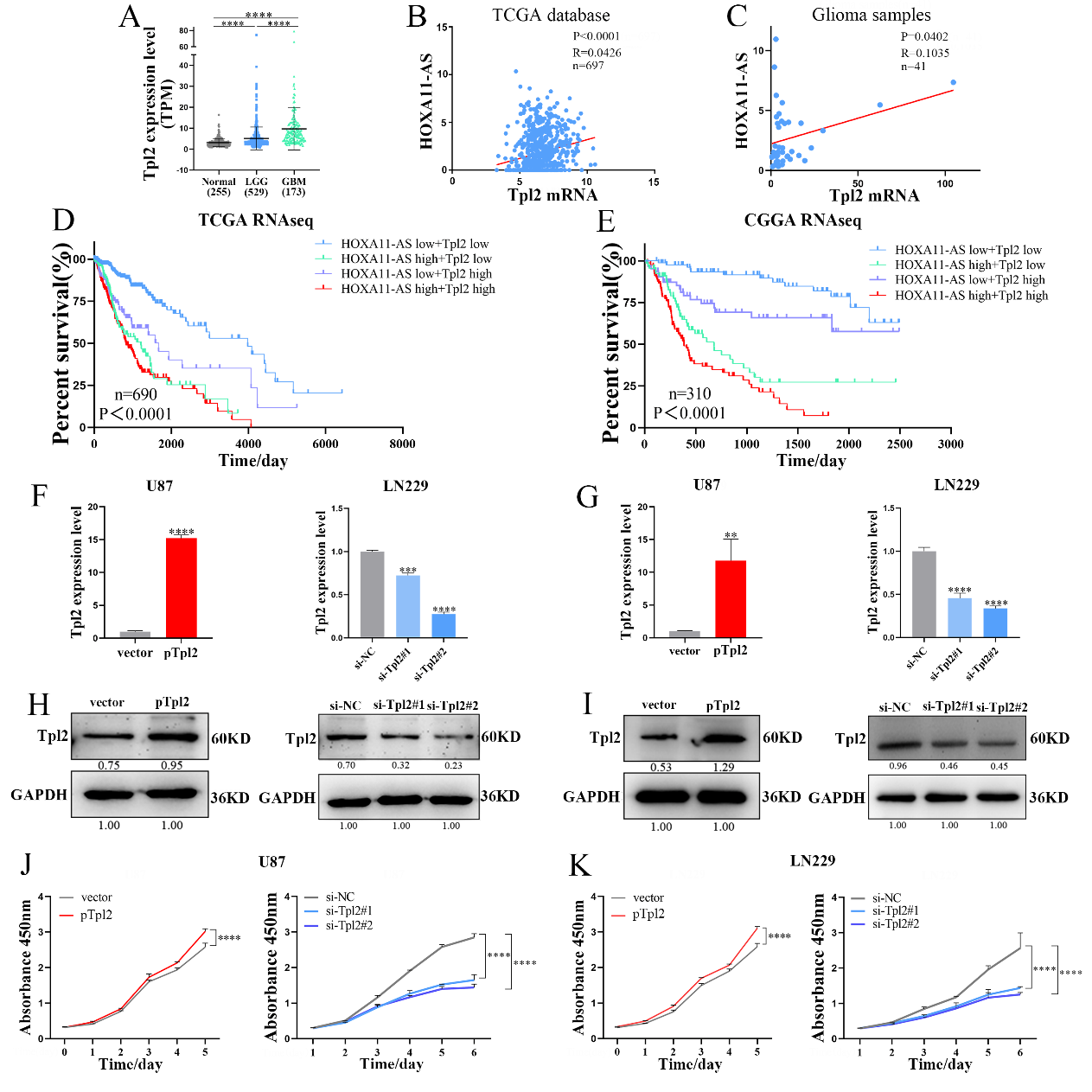


**Figure S13. Clinical features and biological functions of Tpl2 in glioma.**

**(A)** The expression levels of Tpl2 in normal brain tissue, LGG (WHO II-III) and GBM were analyzed via TCGA and GTEx databases. The correlation between Tpl2 mRNA and HOXA11-AS expression level in TCGA database **(B)** and 41 glioma samples **(C)** was analyzed by RT-qPCR. The effect of co-expression of HOXA11-AS and Tpl2 on prognosis was analyzed in TCGA **(D)** and CGGA **(E)** databases. RT-qPCR **(F-G)** and western blot **(H-I)** panels represented Tpl2 expression in U87 and LN229 cells infected with Tpl2 siRNAs or Tpl2 overexpression plasmids (pTpl2). **(J-K)** The effect of Tpl2 on the proliferation of glioma cells was analyzed via CCK-8 experiment. ***P*<0.01, ****P*<0.001, *****P*<0.0001.


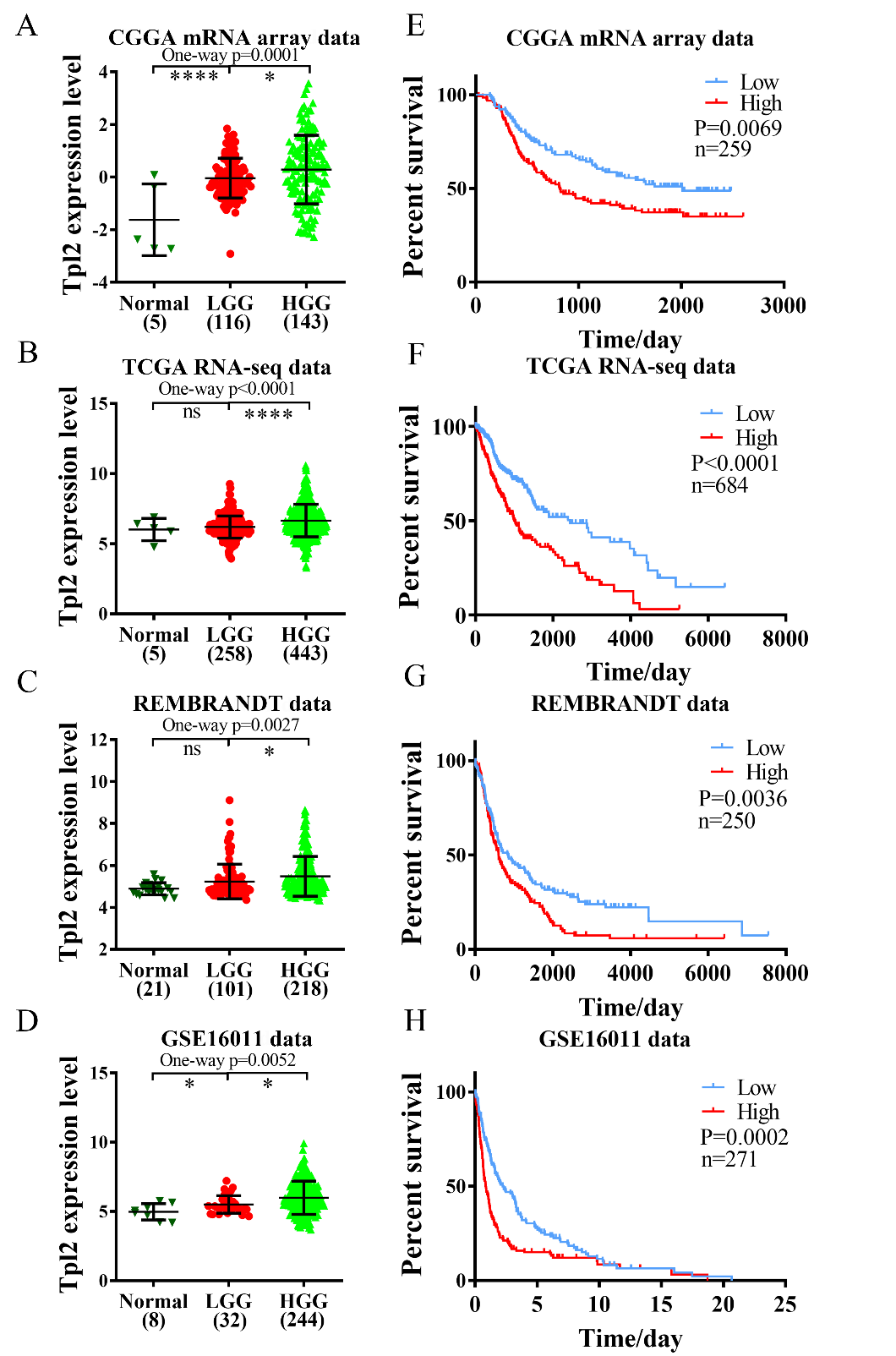


**Figure S14. Correlations between Tpl2 mRNA and grade or prognosis in glioma.**

The expression levels of Tpl2 mRNA within normal tissues, LGG (WHO II) and HGG (WHO III-IV) in CGGA **(A)**, TCGA **(B)**, REMBRANDT **(C)** and GSE16011 **(D)** datasets. Kaplan-Meier survival curves were used to examine the relationships between Tpl2 mRNA expression and the overall survival of glioma patients in the CGGA **(E)**, TCGA **(F)**, REMBRANDT **(G)** and GSE16011 **(H)** datasets. **P*<0.05, *****P*<0.0001.


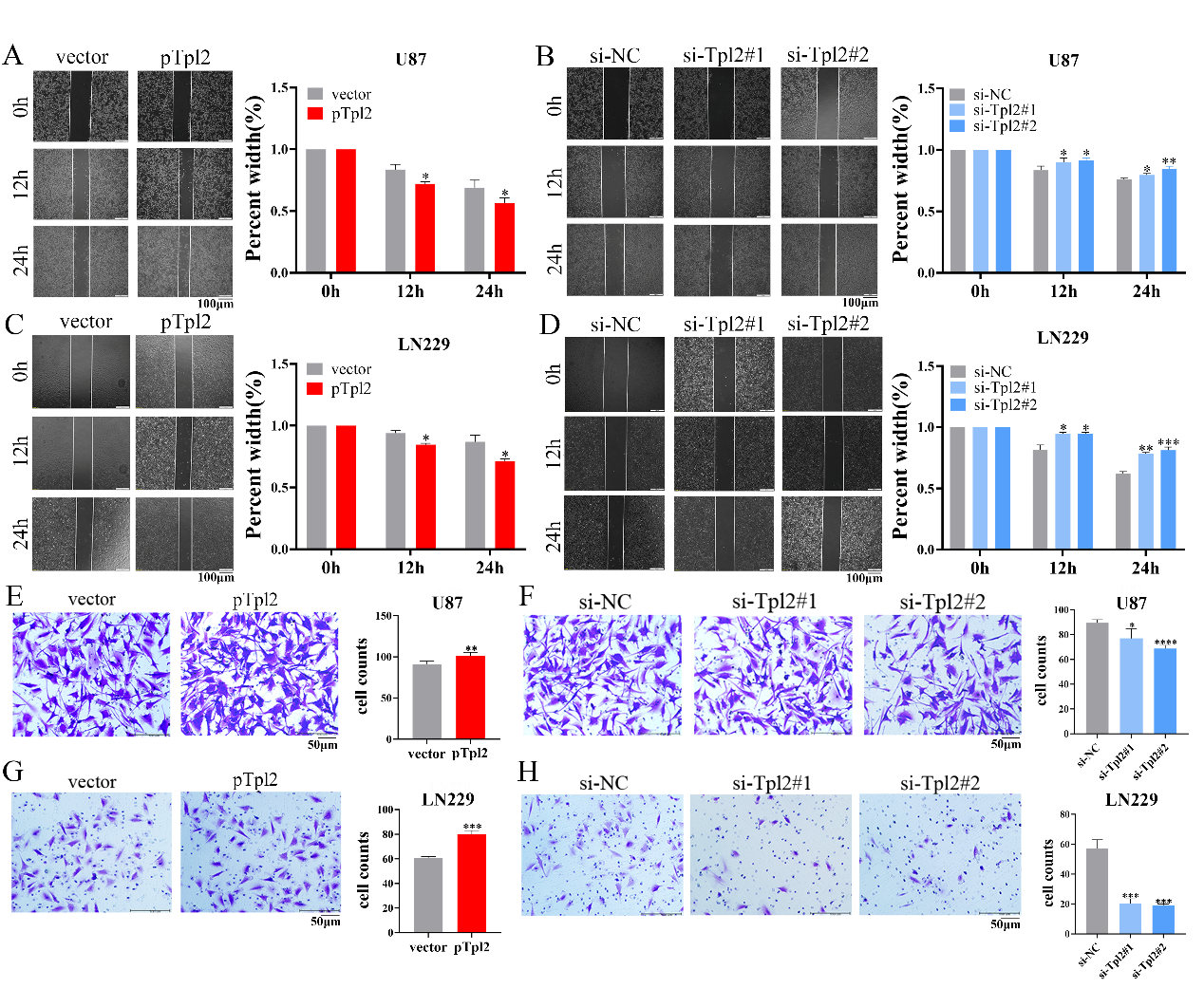


**Figure S15. Tpl2 promoted the migration and invasion of glioma cells.**

**(A-D)** The effect of Tpl2 on glioma cell migration was verified through wound healing experiment. **(E-H)** The effect of Tpl2 on glioma cell invasion was verified through Transwell experiment. **P*<0.05, ***P*<0.01, ****P*<0.001, *****P*<0.0001.


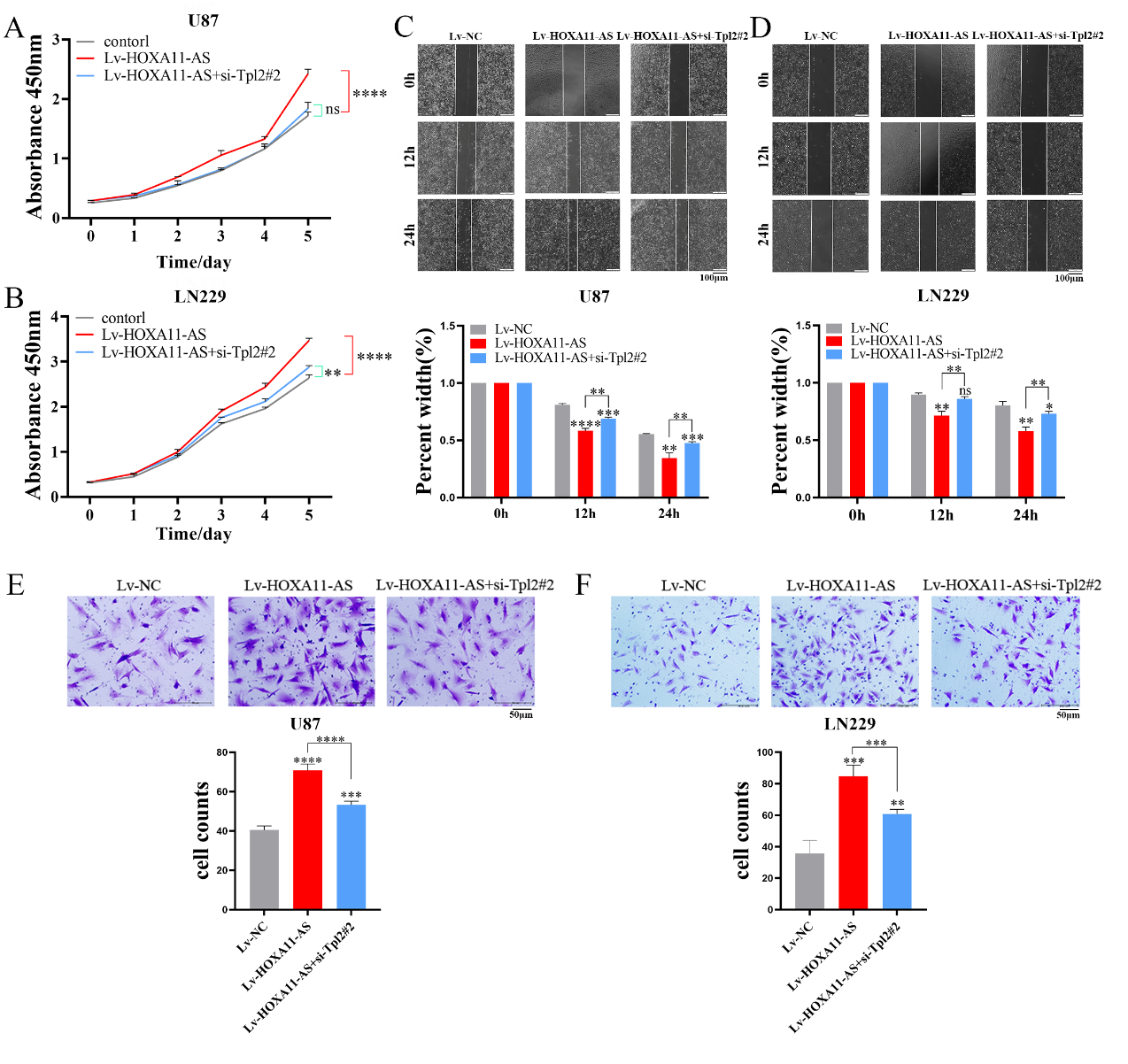


**Figure S16. HOXA11-AS affected the proliferation, migration and invasion of glioma cells by regulating Tpl2.**

**(A-B)** The regulation of HOXA11-AS on glioma cell proliferation depend on Tpl2 was determined via CCK-8 assay. **(C-D)** HOXA11-AS regulating the migration of glioma cells depend on Tpl2 was verified via wound healing experiment. **(E-F)** HOXA11-AS regulating the invasion of glioma cells depend on Tpl2 was confirmed via Transwell experiment. **P*<0.05, ***P*<0.01, ****P*<0.001, *****P*<0.0001.


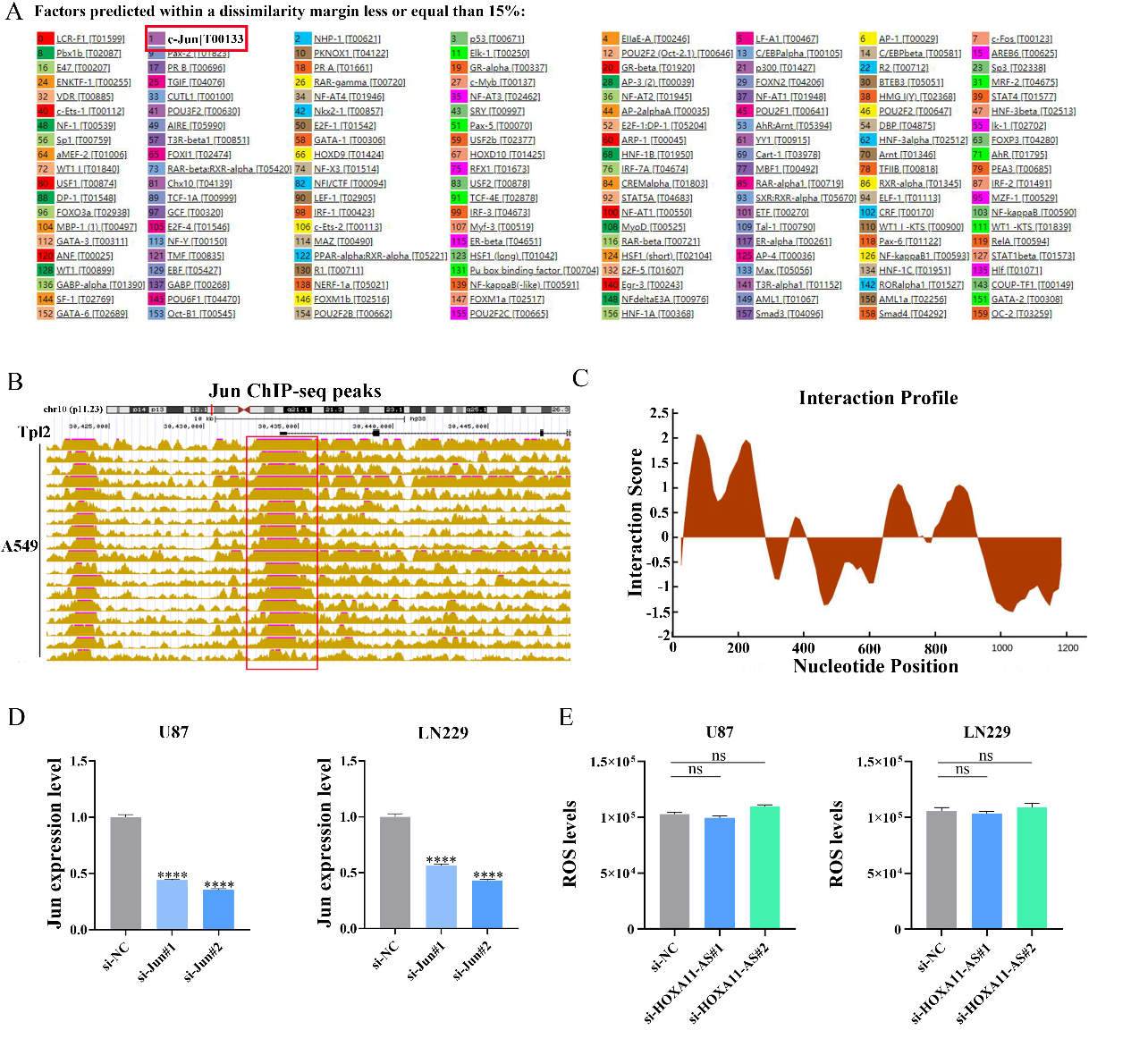


**Figure S17. C-Jun may bind to the Tpl2 promoter region.**

**(A)** Transcription factors that could bind to the Tpl2 promoter region were predicted via the PROMO database. **(B)** The c-Jun binding peaks was detected in the Tpl2 promoter region in A549 cell lines by UCSC database. **(C)** The binding of c-Jun to the Tpl2 promoter region was predicted via Tartaglialab website. **(D)** The c-Jun mRNA expression level after transfected with c-Jun siRNAs was verified in U87 and LN229 cells by RT-qPCR. **(E)** ROS levels in U87 and LN229 cells after HOXA11-AS knockdown were detected by ROS kit. *****P*<0.0001.


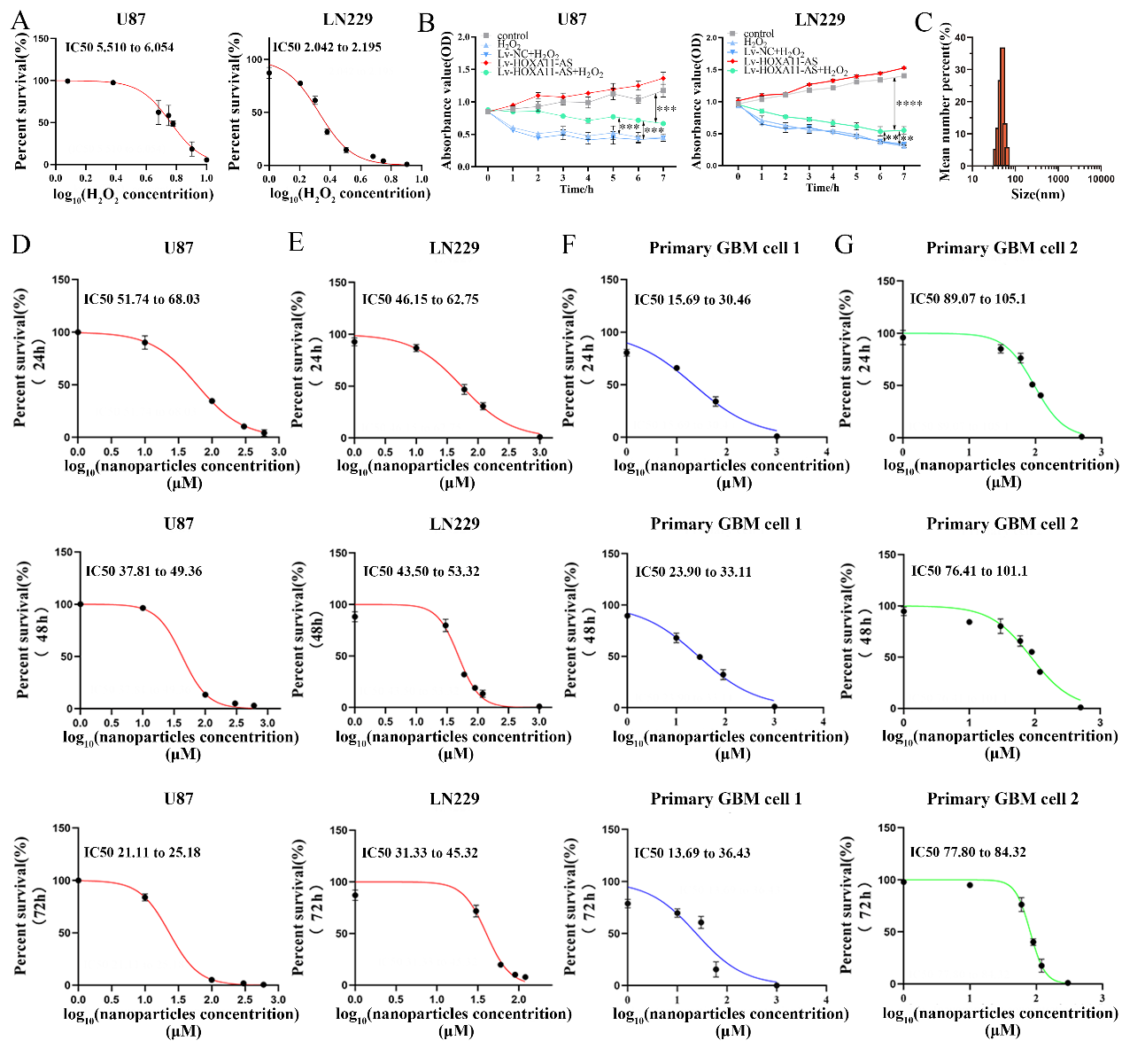


**Figure S18. IC50 validation of** **ROS produced from H_2_O_2_ or nanoparticles (NPs) in U87, LN229 and two patient-derived primary glioblastoma cells.**

**(A)** Detected the concentrations required to cause a loss in cell viability by 50% (IC50) of H_2_O_2_ in U87 and LN229 cells. **(B)** HOXA11-AS reducing the sensitivity of glioma cells to H_2_O_2_ in U87 and LN229 glioma cells was determined by CCK-8 assay. **(C)** The hydrodynamic size distribution of NPs measured by dynamic light scattering (DLS). **(D-G)** IC50 values of ROS produced from nanoparticles (NPs) in U87, LN229, primary glioblastoma cell 1 and primary glioblastoma cell 2 cells, at different times (24h, 48h and 72h) as indicated. The detailed procedure was described in the experimental section. The IC50 values were calculated from dose response curves in three independent experiments. ***P*<0.01, ****P*<0.001, *****P*<0.0001.


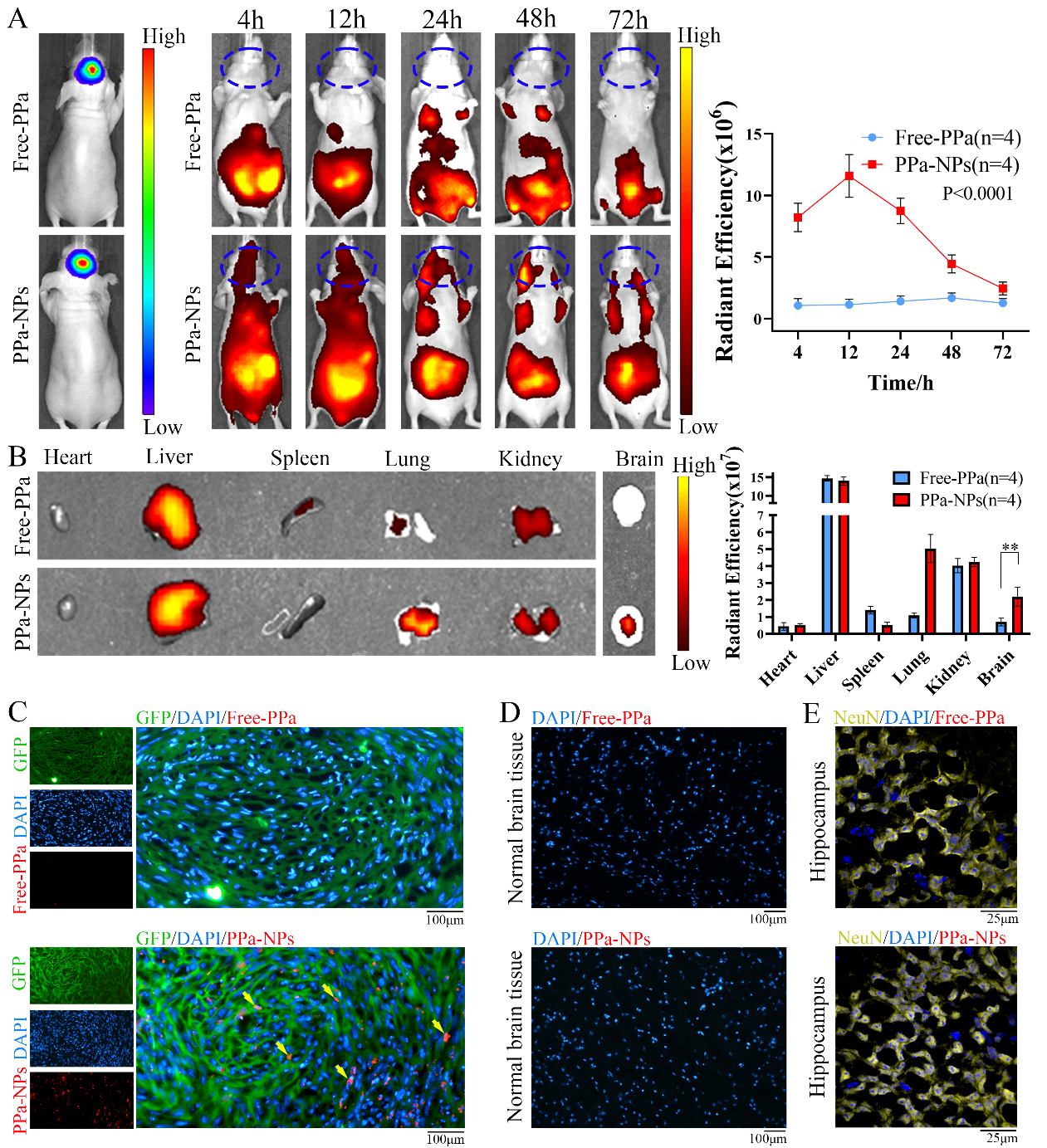


**Figure S19. In vivo evaluation for the glioma-targeting of NPs in orthotopic U87-GFP-luc glioma-bearing mice.**

**(A)** Representative bioluminescence images of glioma-bearing mice at 14 days’ post tumor cell inoculation. The mice were randomly divided into two groups (n=4) and i.v. injected with Free-PPa and PPa-NPs for the subsequent distribution analysis. In vivo distributions and the signal profiles of Free-PPa or PPa-NPs in U87-GFP-luc glioma-bearing nude mice at different time points post i.v. injection. **(B)** Representative ex vivo fluorescence images and corresponding quantitative fluorescence analysis of major organs dissected from glioma-bearing mice at 72 h after i.v. injection with Free-PPa or PPa-NPs. **(C)** Representative fluorescence images of frozen brain sections at 72 h post Free-PPa and PPa-NPs injection showed the effective accumulation of PPa-NPs in glioma as yellow arrows. Representative fluorescence images of frozen brain sections at 72 h post Free-PPa and PPa-NPs injection showed no accumulation of PPa-NPs in normal brain tissue **(D)** and around neurons ([Hippocampus](javascript:;)) **(E)**. ***P*<0.01.


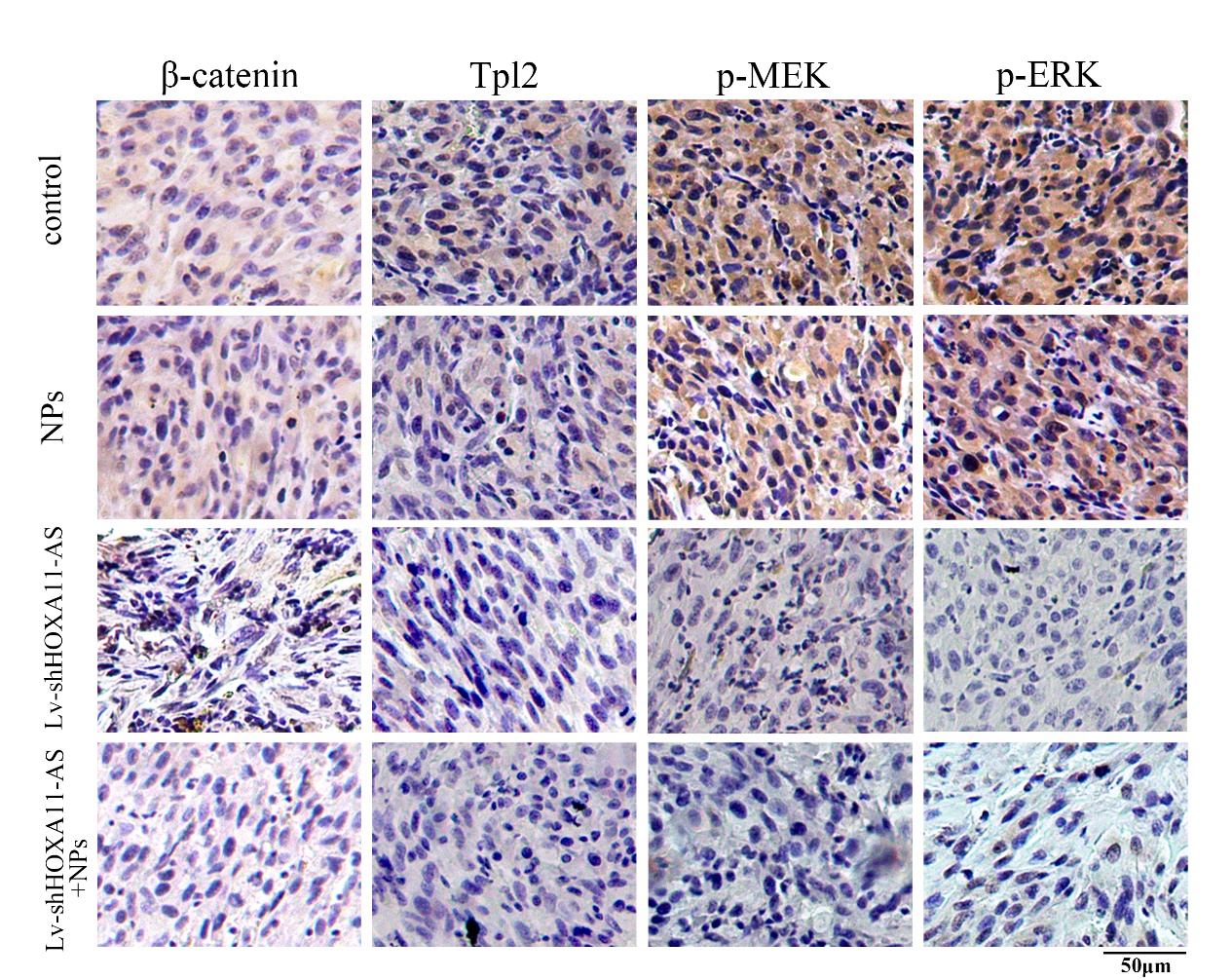


**Figure S20. Images of IHC staining of the glioma of mice bearing orthotopic U87-luc cells treated with PBS, NPs, Lv-shHOXA11-AS and Lv-shHOXA11-AS+NPs.**


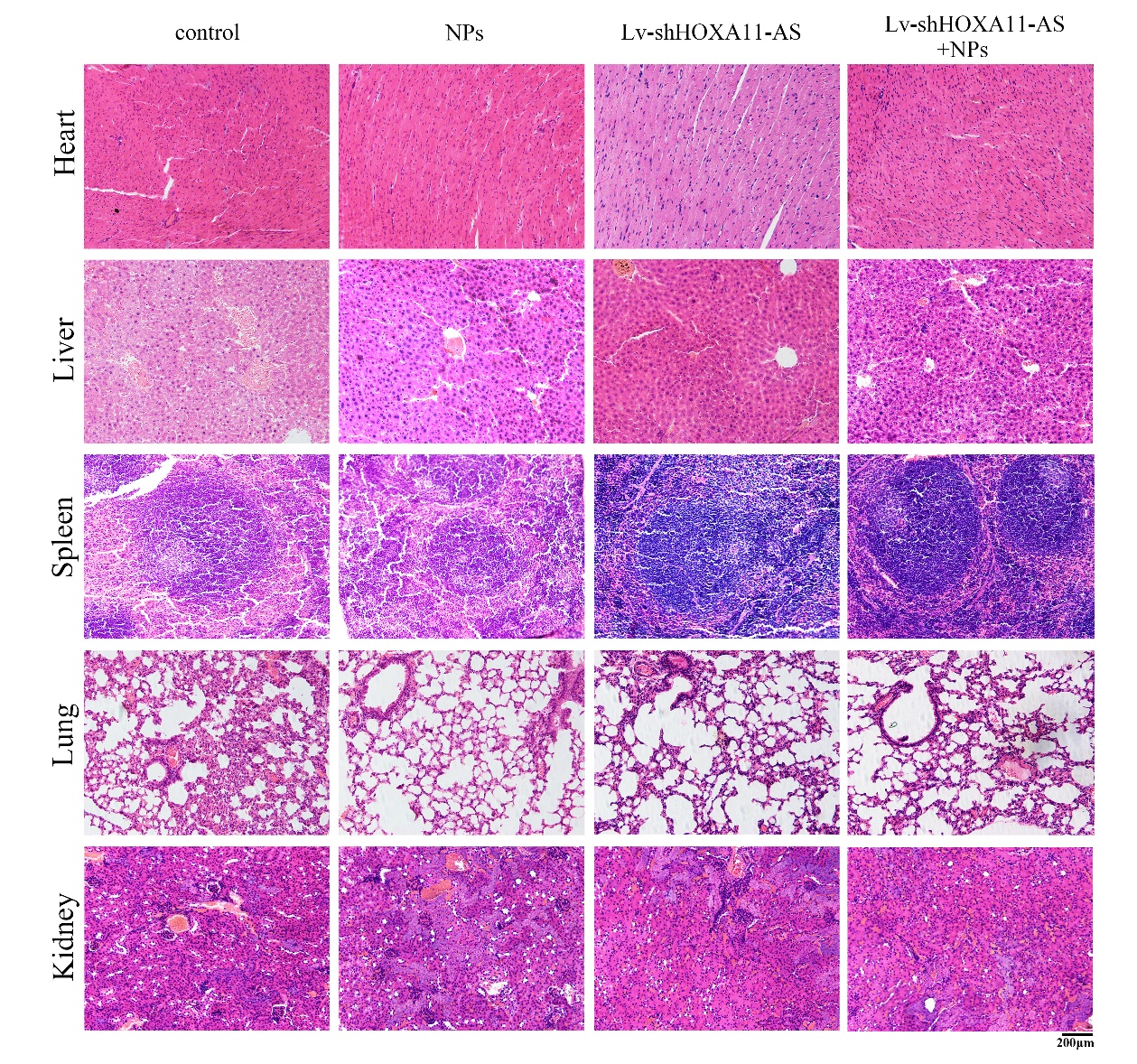


**Figure S21. Images of HE staining of the main organs of mice bearing orthotopic U87-luc cells treated with PBS, NPs, Lv-shHOXA11-AS and Lv-shHOXA11-AS+NPs.**
