# Supplementary material for: LncRNA HOXA11-AS promotes glioma malignant phenotypes and reduces its sensitivity to ROS via Tpl2-MEK1/2-ERK1/2 pathway
Source: Cell Death Dis. 2022 Nov 9;13(11):942. doi: 10.1038/s41419-022-05393-5 (PMC9646708; doi:10.1038/s41419-022-05393-5)

Figure 4F.

U87 and LN229 p-Tpl2


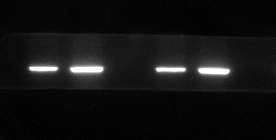


U87 Tpl2


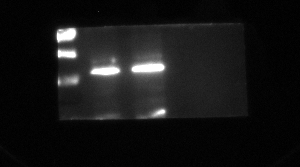


LN229 Tpl2


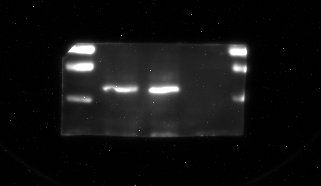


U87 and LN229 p-MEK1/2


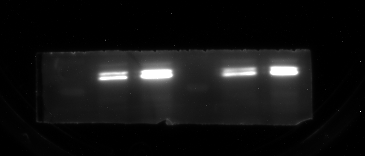


LN229 MEK1/2


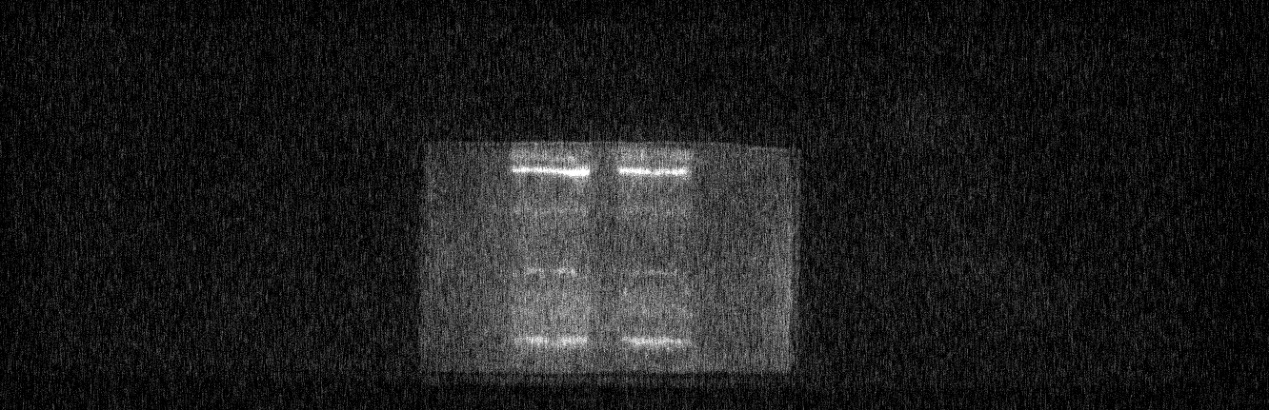


U87 and LN229 p-ERK1/2


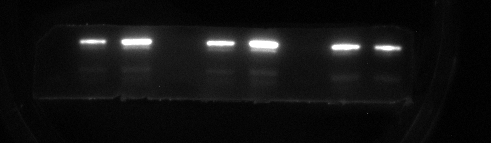


U87 ERK1/2


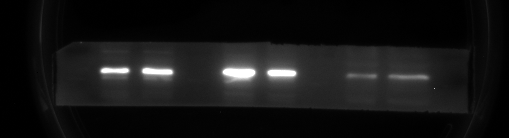


LN229 ERK1/2


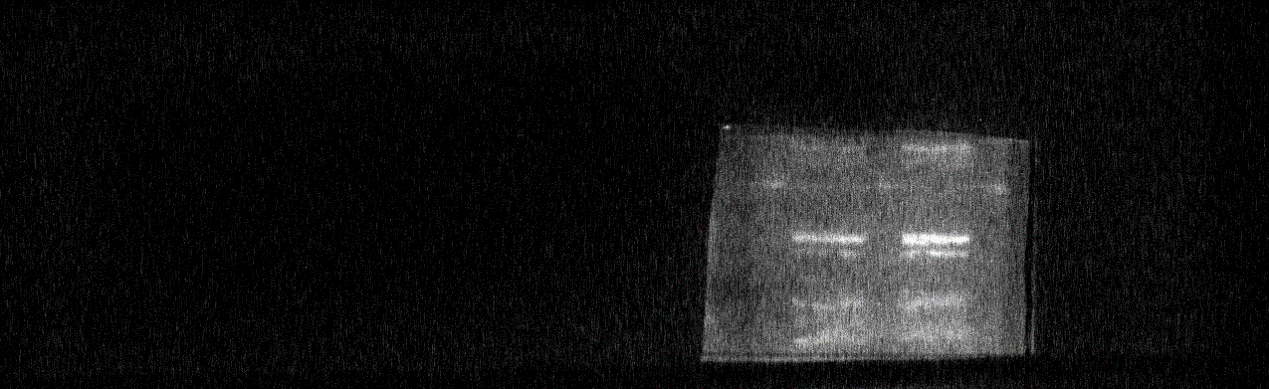


U87 GAPDH


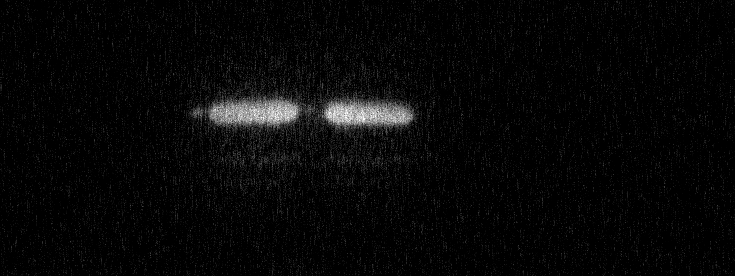


Figure 5J

U87 Tpl2


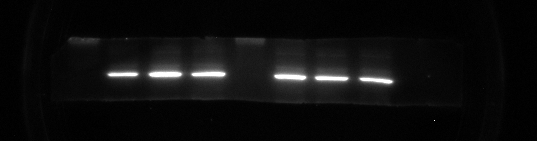


U87 GAPDH


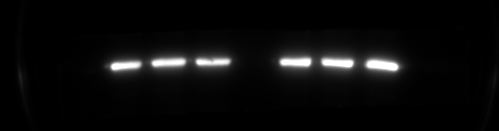


LN229 Tpl2


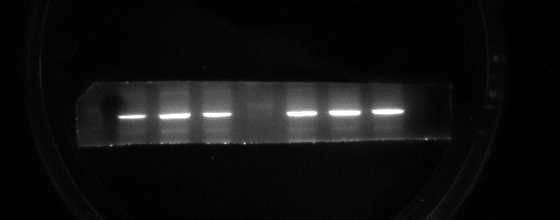


LN229 GAPDH


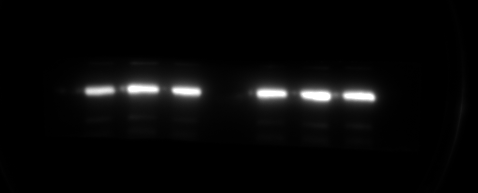


Supplementary Figure 9E

U87 β-catenin(nucleus/cytoplasm/total)


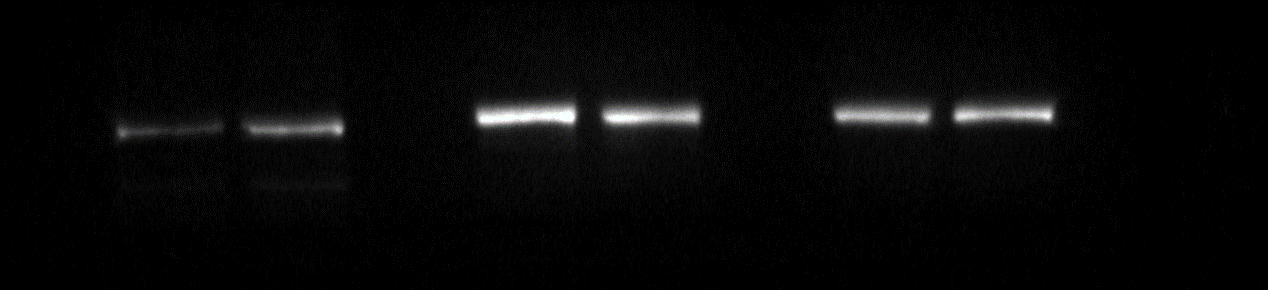


U87 H3(nucleus)


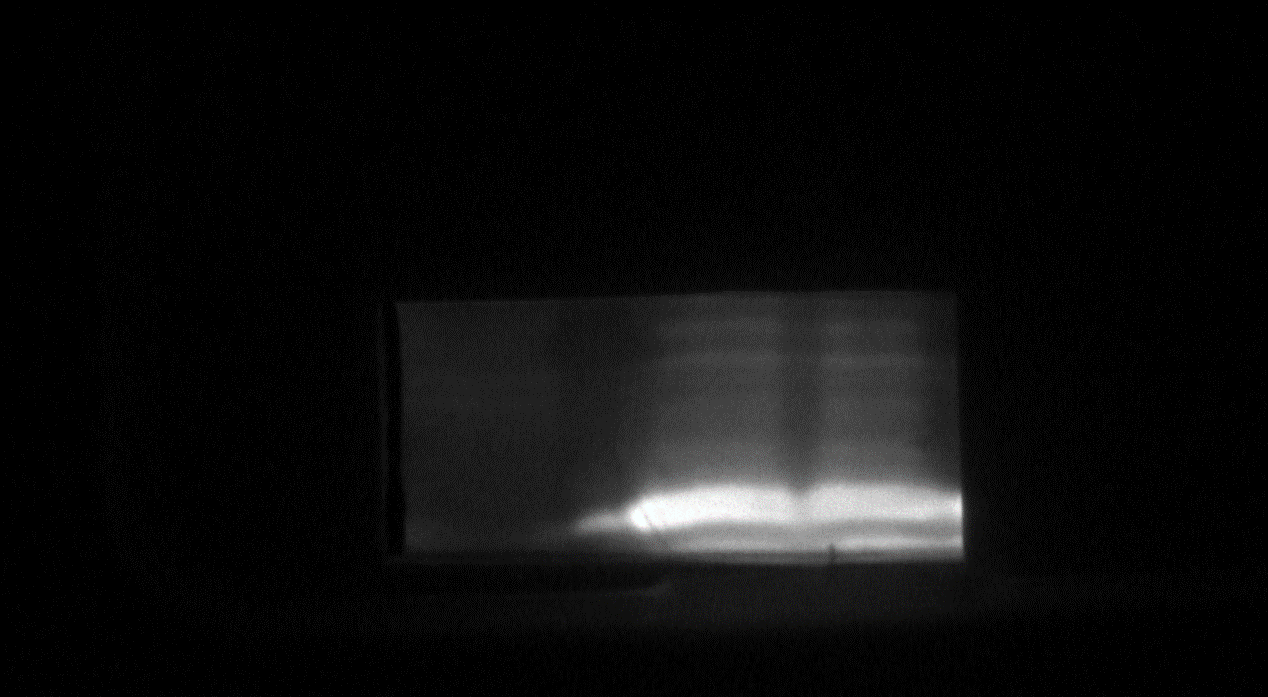


U87 GAPDH (cytoplasm)


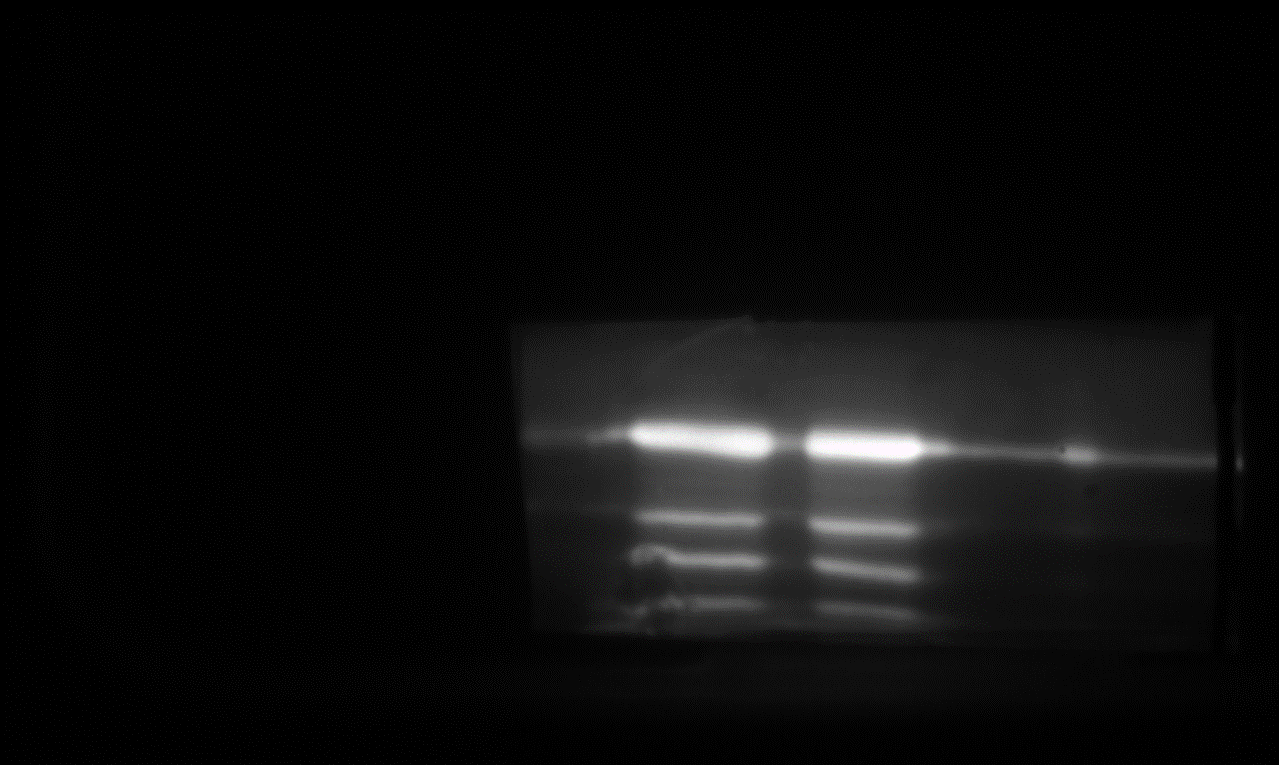


U87 GAPDH (total)


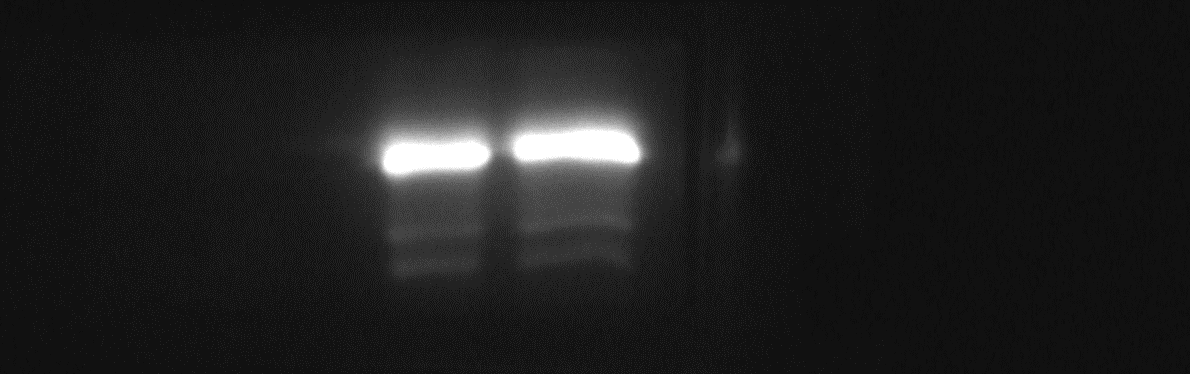


LN229 H3


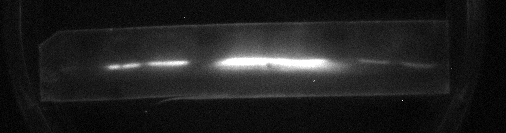


LN229 total GAPDH and cytoplasm GAPDH


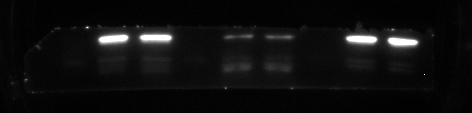


LN229 β-catenin(total/cytoplasm/nucleus)


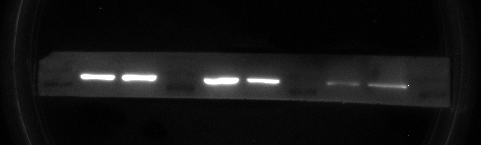


Supplementary Figure 13H

U87 pTpl2 and si-Tpl2


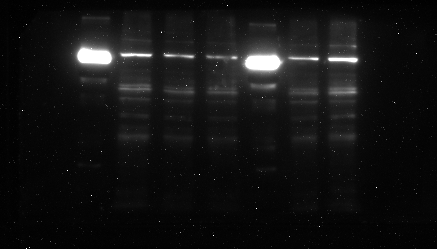


Supplementary Figure 13I

LN229 pTpl2


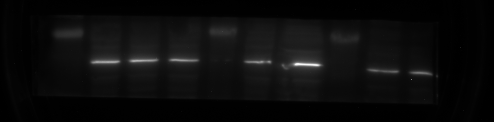


LN229 si-Tpl2


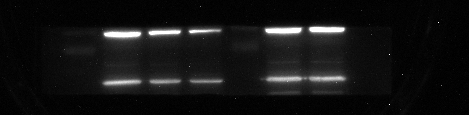

Supplement: Supplementary file 10 — Original full length western blots [file 41419_2022_5393_MOESM10_ESM.docx]
